# Mosaic Inverted Hemagglutinin Extracellular Vesicle Vaccines Elicit Protective Systemic and Mucosal Immunity against Heterosubtypic Influenza Infection

**DOI:** 10.1021/acsnano.5c13363

**Published:** 2026-04-01

**Authors:** Wandi Zhu, Lai Wei, Chunhong Dong, Joo Kyung Kim, Madeline Bruhn, Yao Ma, Alex Ferrante, Arini Arsana, Priscilla Omotara, Sang-Moo Kang, Bao-Zhong Wang

**Affiliations:** Center for Inflammation, Immunity & Infection, Institute for Biomedical Sciences, 1373 Georgia State University, Atlanta, Georgia 30303, United States

**Keywords:** extracellular vesicle, mosaic HA vaccine, influenza virus, intranasal immunization, mucosal immune response, HA stalk, cross-protection

## Abstract

Developing innovative vaccine platforms and delivery strategies to induce broad, protective immunity in the respiratory tract is crucial for preventing influenza infection and transmission in potential epidemics and pandemics. In this study, we used cell-derived extracellular vesicles (EVs) as a vaccine platform to display mosaic human and avian influenza hemagglutinins (HAs) concurrently, including HA1/HA9/HA12 or HA3/HA4/HA10, on the EV surfaces. Immunization with the mosaic HA-EV vaccine elicited cross-reactive antibodies against influenza HA stalks and viruses, robust virus-specific cellular immune responses, and a balanced Th1/Th2 immune profile. Notably, the EVs demonstrated a promising application as an effective mucosal vaccine strategy, as evidenced by enhanced HA stalk- and virus-specific IgA in mucosal tissues and complete protection against heterosubtypic reassortant H7N9 and H5N1 virus infections in mice via intranasal immunization. EV-based mosaic HA vaccines hold great promise for developing universal influenza vaccines that target a mucosal route.

## Graphical abstract

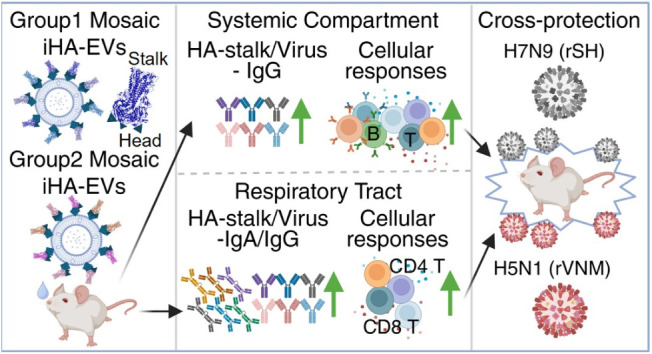

Mucosal vaccination effectively induces local immune responses, protecting against respiratory virus infections at the site of invasion. Although various mucosal vaccine formulations with innovative delivery and adjuvant systems have been studied for intranasal administration against respiratory virus infections in clinical trials, FluMist (MedImmune and AstraZeneca) remains the only FDA-approved mucosal influenza vaccine.^1,2^ The mucosal vaccine clinical trials were mainly based on live-attenuated virus or adenovirus vector vaccines.^3–5^ However, live-attenuated vaccines have potential hazards for immunodeficient and immunosuppressed individuals, and preexisting antiadenovirus immunity can significantly reduce the efficacy of adenovirus vector vaccines.^6^ Researchers have endeavored to develop alternative viral vectors, such as Parainfluenza virus 5 (PIV5)^7,8^ and murine pneumonia virus (MPV),^9,10^ and have evaluated them in clinical trials for intranasal applications. These clinical trials will provide evidence on the safety and efficacy of the viral vector vaccines in humans. Developing an effective mucosal vaccination strategy that elicits robust mucosal immune responses while minimizing safety concerns remains urgently needed.

Cell-derived extracellular vesicles (EVs) are natural nanoparticles that facilitate cell-to-cell communications. EVs have been investigated and engineered for vaccine and drug delivery due to their biocompatibility and safety.^11,12^ Exosomes and ectosomes are two classes of EVs under investigation for their potent applications in disease diagnosis and therapy.^13^ Exosomes originate as intraluminal vesicles (ILVs) within multivesicular bodies (MVBs) and are released when MVBs fuse with the plasma membrane. In contrast, ectosomes form through the direct outward budding and shedding of the plasma membrane.^14^ Our recent study demonstrated the significant promise of EVs as a vaccine platform and adjuvant for intranasal vaccines.^15,16^ Different cell-derived EVs exhibited potent adjuvanticity by preferentially promoting the activation and maturation of innate immune cells. Intranasal immunizations of influenza hemagglutinin (HA)-conjugated EVs induced strong immune responses in both systemic compartments and the respiratory tract and conferred cross-protection against heterologous influenza infection.^15^ In addition, we demonstrated that matured dendritic cell-derived EVs could be used as a mucosal adjuvant in combination with recombinant HA protein, significantly enhancing immune responses and protection efficacy following intranasal delivery.^16^ The Th1 and Th2 preference depends on the vaccine categories, delivery routes, supplemented adjuvants, and immunization strategies. Vaccines that stimulated strong and balanced Th1/Th2 immune responses provided improved protection compared to Th-biased immune responses.^17–19^ Like the immune phenotypes induced by mRNA lipid nanoparticle vaccines, EV-based vaccines effectively stimulated Th1 and Th2 immune responses while offering safety advantages for intranasal administration.^15,20^ Although EV platforms have demonstrated safety profiles in different studies, the unfavorable immunogenicity of EVs remains largely unexplored.^21^ Repeated vaccination may elicit EV-directed immune responses that influence the efficacy of subsequent vaccinations using the same platform. Future studies will be needed to evaluate anti-EV immunity and its effect on boosting vaccinations.

The selection of viral antigens is crucial in vaccine design to improve protective efficiency. Compared to the highly variable HA head, the conserved HA stalk domain has emerged as a promising candidate for a universal influenza vaccine due to its low evolutionary rate and greater tolerance to mutations.^22,23^ Broadly neutralizing antibodies specific to the HA stalk have been identified in individuals infected with influenza, indicating that the human immune system is capable of recognizing this conserved region.^24–26^ Although previous clinical trials of chimeric HA-based universal influenza vaccines yielded limited success,^23,27,28^ Phase 1 trials based on a self-assembled ferritin nanoparticle platform displaying H2 HA, stabilized H1 stem (H1ssF), and H10ssF successfully elicited broadly neutralizing antibody responses against the conserved HA stem in healthy adults.^29–31^ Furthermore, a clinical trial (NCT05755620) evaluating the safety and immunogenicity of H1ssF-3928 mRNA-LNP vaccine highlights the potential of novel approaches to induce HA stalk-specific antibody responses and improve balanced Th1/Th2 immune activation for the development of a universal influenza vaccine. However, the recombinant HA stalk domain may lose critical conformational integrity during HA reconstruction. Our findings suggest that utilizing the entire HA ectodomain as an immunogen, while hiding the HA head and increasing exposure of the HA stalk, is an effective strategy to induce robust immune responses targeting conserved HA epitopes.^15^

Mosaic vaccine strategies that elicit broad-spectrum immune responses and cross-protection are important for advancing universal vaccines against influenza and SARS-CoV-2.^32–36^ Influenza HA plays an essential role in virus invasion by mediating adhesion between its head domain and sialic acid residues on the host cell surface. We have leveraged the HA-sialic acid interaction to display the HA-stalk domain on the surfaces of EVs.^15^ In this study, we developed EV-based mosaic influenza vaccines by displaying multiple HAs in an inverted configuration on the EV surface, thereby enhancing antibody responses targeting the most conserved sites of HA stalks and heads. Intranasal administration of the EV-based mosaic influenza vaccine elicited strong, broadly reactive humoral and cellular immune responses in systemic compartments and generated robust mucosal immunity in the respiratory tract. These responses targeted HA stalks and a diverse range of influenza strains spanning both groups of influenza A viruses, which provided complete cross-protection against heterosubtypic reassortant H7N9 and H5N1 influenza viruses in mice.

## Results

### Generation and Primary Immunogenicity of Extracellular Vesicles (EVs) Displaying Mosaic Inverted HAs

To develop a mosaic EV-based influenza vaccine with broad protection against diverse strains, we engineered EVs displaying three different HA proteins from each of the two HA phylogenetic groups (HA1/HA9/HA12 from Group 1 and HA3/HA4/HA10 from Group 2). This design exposes the HA-stalk domains while partially concealing the immunodominant HA heads, unlike the native HA on the influenza virus particle. The schematic diagram of Group 1 mosaic HA-EVs, Group 2 mosaic HA-EVs, and the influenza virus was displayed in Figure 1A. As shown in Figure 1B, compared to single HA-EVs and the soluble HA3 protein, the mosaic G1 and G2 HA-EVs displayed multiple, broadened bands around the expected HA molecular weight in Coomassie blue staining and Western blot analysis, indicating the presence of different HA proteins within the mosaic EV particles. The classical exosome markers Alix, TSG101, and CD81 were detected in both mosaic HA-EVs and control EVs by Western blot analysis (Figure 1B). Dynamic Light Scattering (DLS) analysis and transmission electron microscopy (TEM) imaging were used to characterize the size distribution and morphology of mosaic Group 1 and Group 2 HA-EVs (Figure 1C and D). The EVs generated in this study exhibited a broad size distribution, indicating a heterogeneous population comprising both exosomes and ectosomes. CR9114 is a broadly neutralizing antibody that targets the conserved HA stalk and confers protection against both influenza A and B viruses.^37^ ELISA analysis demonstrated that mosaic HA-EVs were recognized by CR9114 in a dose-dependent manner (Figure 1E), indicating that the HA stalk region was presented in its native conformation on the surface of HA-EVs. To evaluate the immunostimulatory activity of mosaic HA-EVs *in vitro*, bone marrow-derived dendritic cells (BMDCs) were cultured and stimulated with mosaic HA-EVs. Monophosphoryl lipid A (MPLA) was included as a control. Compared with unstimulated cells, both mosaic HA-EVs and MPLA significantly promoted BMDC maturation, as evidenced by increased expression of CD40 and CD86. Meanwhile, mosaic HA-EVs induced higher levels of TNF-α and IL-12/p70 in culture supernatants than MPLA (Figure 1F).

**Figure 1:**
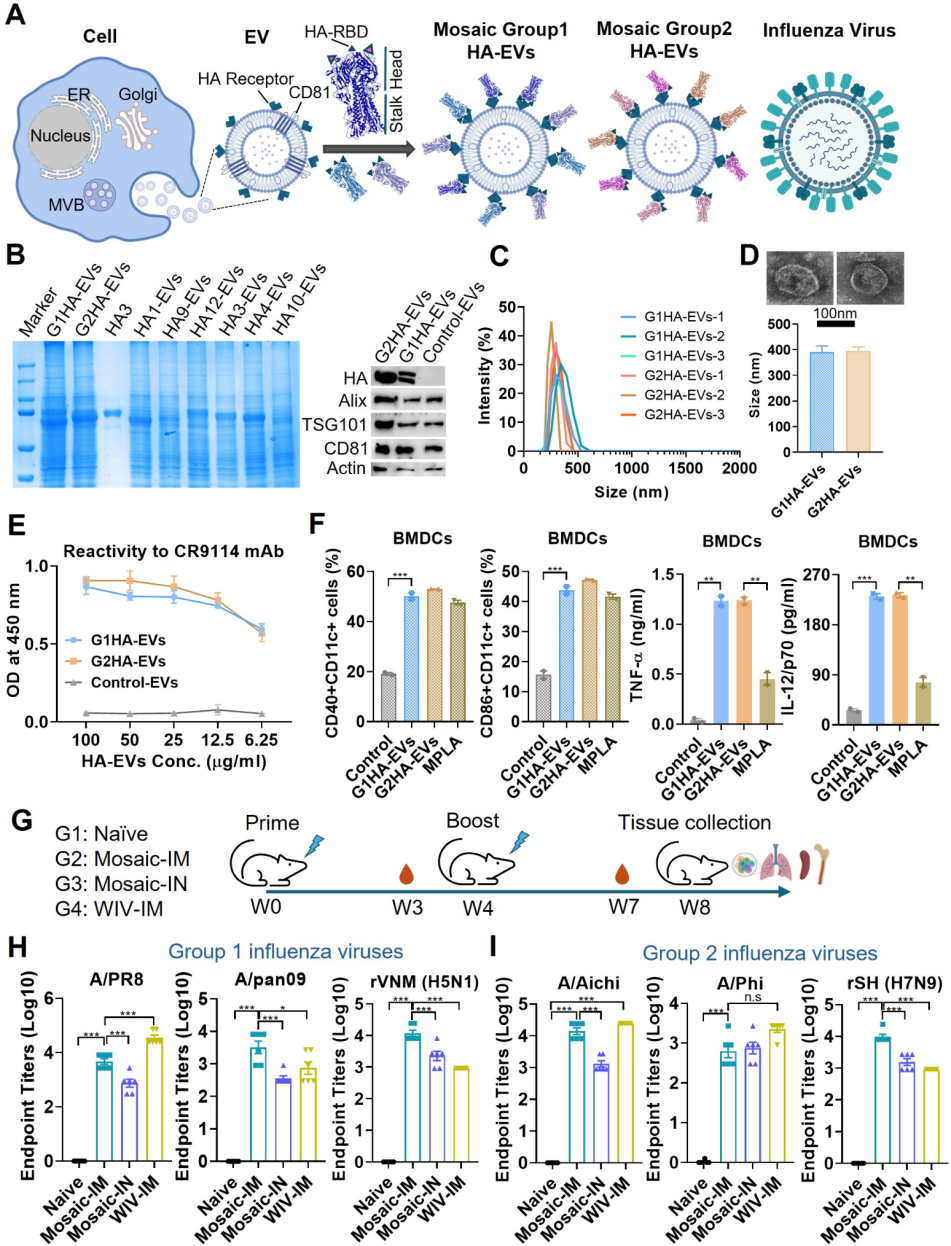
Generation of mosaic inverted HA EV vaccine. (A) The schematic diagram of EV-based mosaic vaccines. Different influenza HAs from Group 1 and Group 2 influenza viruses were conjugated to the EV surface via HA-sialic acid receptor binding to generate the Mosaic Group 1 HA-EVs and Mosaic Group 2 HA-EVs vaccines, respectively. (B) Analyze the content of HA and the expression of classical EV makers in the mosaic vaccines by Coomassie blue staining and Western blot. (C) and (D) The size distribution and morphology of the Group 1 and Group 2 HA-EVs by DLS and TEM imaging. (E) Recognition of HA-EVs by HA stalk-specific monoclonal antibody CR9114. (F) Activation of BMDCs by mosaic HA-EVs. (G) Immunization and sample collection diagram: mice were intramuscularly or intranasally immunized with EVs-based mosaic HA-stalk vaccines (Mosaic-IM and Mosaic-IN), and the mice that received bivalent whole inactivated virus vaccines (WIVs-IM) were included as controls. The mouse sera were collected at 3 weeks post-immunization for analysis. (H) and (I) Different viruses specific IgG in prime sera. *P* < 0.05 (*), *P* < 0.01 (**), *P* < 0.001 (***), *P* > 0.05 (n.s.).

Tetraspanin CD81 is a classical scaffold molecule on EVs.^38^ We found that overexpression of CD81 tremendously increased EV yield in cell cultures (Figure S1). DLS analysis showed a similar size distribution of CD81-overexpressed EVs compared to control EVs (Figure S1A). Overexpression of CD81 resulted in enhanced EV secretion with increased particle numbers and total protein amount, as determined by TEM, nanoparticle tracking analysis (NTA), and BCA assays (Figure S1B and C). These results suggest that modifications to the cell culture system can significantly enhance EV yields, which could be used for large-scale production of EV-based vaccines.

To evaluate the immunogenicity of the mosaic HA-EVs *in vivo*, 6- to 8-week-old BALB/c mice were immunized intramuscularly (Mosaic-IM) or intranasally (Mosaic-IN) with mixed mosaic HA-EVs. Control groups included naïve mice and those immunized intramuscularly with whole inactivated viruses (WIV-IM) from A/Aichi (H3N2) and A/PR8 (H1N1) (Figure 1G). Sera collected 3 weeks after the primary immunization showed that both the Mosaic-IM and Mosaic-IN groups exhibited significantly increased antigen-specific IgG levels against purified HA proteins (HA1, HA9, HA12, HA3, HA4, and HA10) from both HA groups. In contrast, the WIV-IM immunization primarily induced HA1- and HA3-specific IgG (Figure S2A). Mosaic-IM immunization further stimulated cross-reactive IgG response against heterosubtypic reassortant H5N1 (rVNM) and H7N9 (rSH) but showed no significant advantage over WIV-IM in inducing antibodies against homologous (A/PR8, A/Aichi) and heterologous (A/pan09 H1N1, A/Phi H3N2) viruses. The Mosaic-IN group induced moderate virus-specific IgG levels compared to the intramuscularly immunized groups (Figures 1H,I, and S2B). The inconsistent responses to HA proteins and viruses suggest that the viral components besides HA in WIV formulations contribute to cross-reactive antibody responses against heterologous influenza viruses. In brief, EV-based mosaic inverted HA vaccines demonstrated promising immunogenicity through both IM and IN routes, offering potential applications for broad influenza protection.

### Mosaic Inverted HA-EVs Induced HA Stalk- and Broad Virus-Specific Antibody Responses

Sera were collected 3 weeks post-boost vaccination to evaluate humoral immune responses. Mosaic-IM and Mosaic-IN boosting vaccinations stimulated robust IgG, IgG1, and IgG2a antibody responses against homologous (A/Aichi and A/PR8), heterologous (A/Phi and A/pan09), and heterosubtypic (rSH and rVNM) influenza strains spanning the two influenza A virus groups (Figures 2 A,B,C, and S3). Compared to the WIV-IM group, which failed to induce HA stalk-specific antibodies, Mosaic-IM and Mosaic-IN significantly increased HA stalk-specific IgG, IgG1, and IgG2a (Figure 2D and E). In addition, these vaccines preferentially induced strong virus- and HA stalk-specific IgG1 and IgG2a responses, resulting in a relatively balanced IgG1/IgG2a ratio (Figure 2F). In contrast, recombinant protein and protein nanoparticle vaccines predominantly stimulate IgG1-biased, Th2-skewed immune responses, necessitating the use of adjuvants or additional modifications to redirect Th immune phenotypes.^15,18^ These findings suggest that the EV vaccine platform effectively delivers immunological signals to promote both Th1- and Th2-type immunity without requiring adjuvant supplementation. Neutralization and hemagglutination inhibition (HAI) assays were performed to determine neutralizing and HAI titers in post-boost sera. Both i.m. and i.n. immunization with the mosaic HA-EV vaccine induced neutralizing titers (50% inhibitory concentration, IC50) and HAI titers ≥ 40, suggesting potential protective efficacy against A/Aichi (H3N2) and A/PR8 (H1N1) viruses. The WIV-IM group exhibited the highest neutralizing and HAI titers against homologous viruses (Figure 2G and H). In addition, sera from the mosaic HA-EV and WIV-IM groups demonstrated neutralizing ability against the heterologous A/Phi (H3N2) virus. Notably, mosaic HA-EV immunization elicited higher IC50 values against rSH and rVNM compared with WIV-IM (Figure 2I). Furthermore, sera from mosaic HA-EV-immunized mice partially inhibited CR9114 binding to mosaic HA-EVs, whereas sera from the WIV-IM group did not (Figure 2J). These findings indicate that mosaic HA-EV immunization elicited HA stalk-specific antibodies targeting epitopes overlapping with those recognized by CR9114.

**Figure 2:**
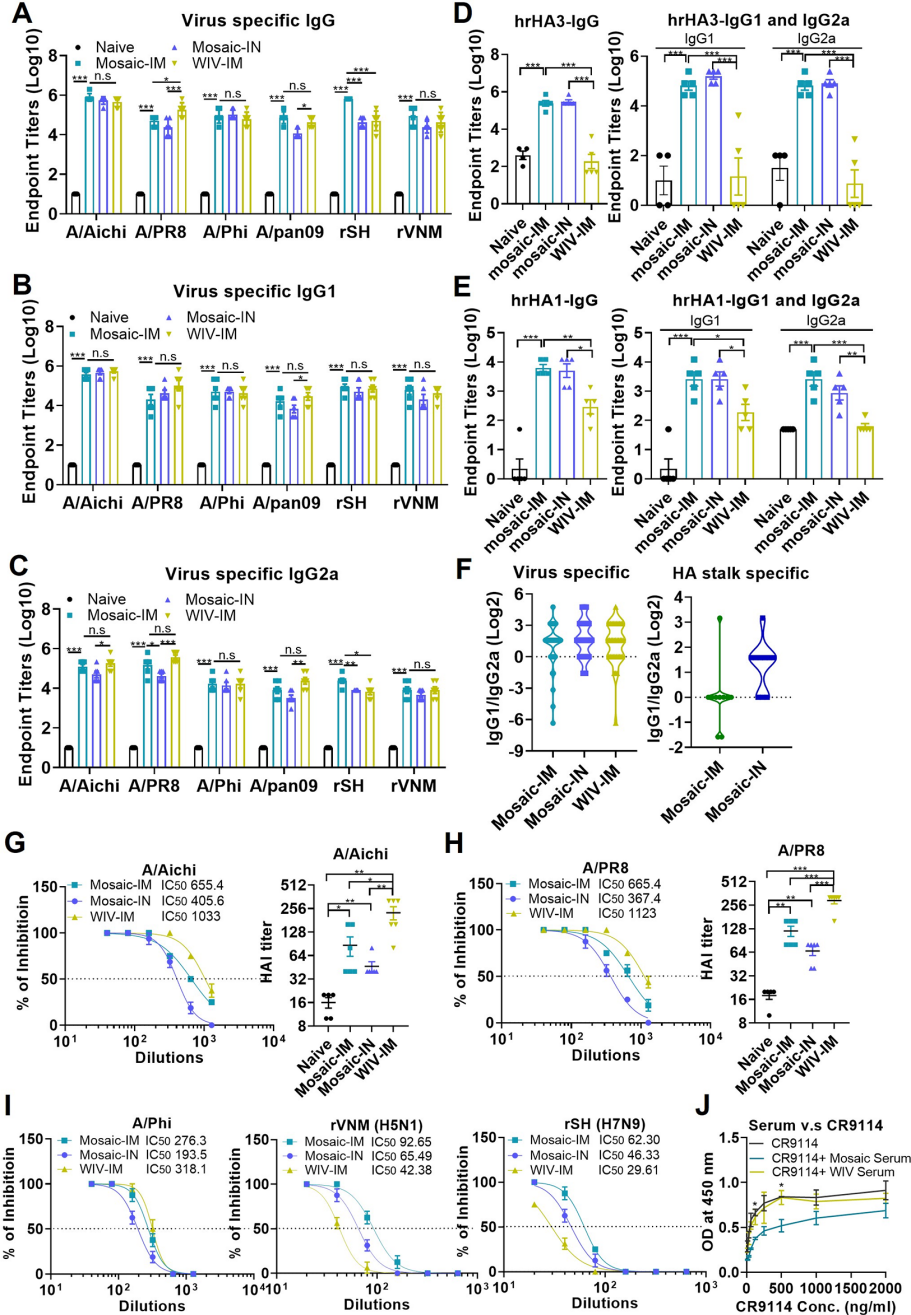
Mosaic inverted HA EV vaccines induced cross-reactive antibody responses against viruses and HA stalks. Mouse sera were collected 3 weeks post-boost immunization for analysis of antibody responses. A/Aichi, A/PR8, A/Phi, A/pan09, rSH, and rVNM virus-specific IgG (A), IgG1 (B), and IgG2a (C). (D) and (E) HA stalk-specific IgG and IgG isotypes. (F) The ratios of IgG1 to IgG2a. (G) and (H) Neutralizing and HAI titers against A/PR8 and A/Aichi viruses. (I) Neutralization titers against A/Phi, rSH, and rVNM. (J) Inhibition of CR9114 mAb binding by immune sera. *P* < 0.05 (*), *P* < 0.01 (**), *P* < 0.001 (***), *P* > 0.05 (n.s.).

### Systemic Cellular Immune Responses Induced by Mosaic HA EV Vaccines

To determine cellular immune responses, mice were euthanized 4 weeks post-boosting vaccination for spleen and bone marrow (BM) collection. Mosaic-IM and Mosaic-IN vaccinations increased the number of HA3-specific antibody-secreting cells (ASCs) in both the spleen and BM, while WIV-IM predominantly induced HA3-specific ASCs in BM (Figure 3A and B). As expected, antibodies secreted by the spleen and BM cells from all immunized groups recognized the homologous A/Aichi and A/PR8 viruses. However, Mosaic-IM uniquely induced ASCs specific to heterologous (A/Phi and A/pan09) and heterosubtypic (rSH and rVNM) viruses compared to Mosaic-IN and WIV-IM (Figure 3C and D). Both mosaic EVs and WIV elicited significant numbers of IL-4- and IFN-γ-secreting cells in the spleen after stimulation with homologous, heterologous, or heterosubtypic influenza viruses (Figure 3E,F). These results are consistent with the increased IgG1 and IgG2a levels observed in Figure 2, further demonstrating the role of the EV platform in harmonizing Th1/Th2 immune responses. Interestingly, Mosaic-IM and Mosaic-IN stimulated higher numbers of H3 and H7 peptide-specific IL-4- and IFN-γ-secreting cells than naïve and WIV-IM groups (Figure 3G and H). The increased rSH- and rVNM-specific IL-4- and IFN-γ-secreting cells observed in WIV-IM vaccinations may predominantly target other conserved viral antigens, such as nucleoprotein, polymerase complex, or matrix protein. Therefore, these findings highlight the potential of mosaic inverted HA-EVs as effective vaccine candidates to induce broad systemic humoral and cellular immunity.

**Figure 3:**
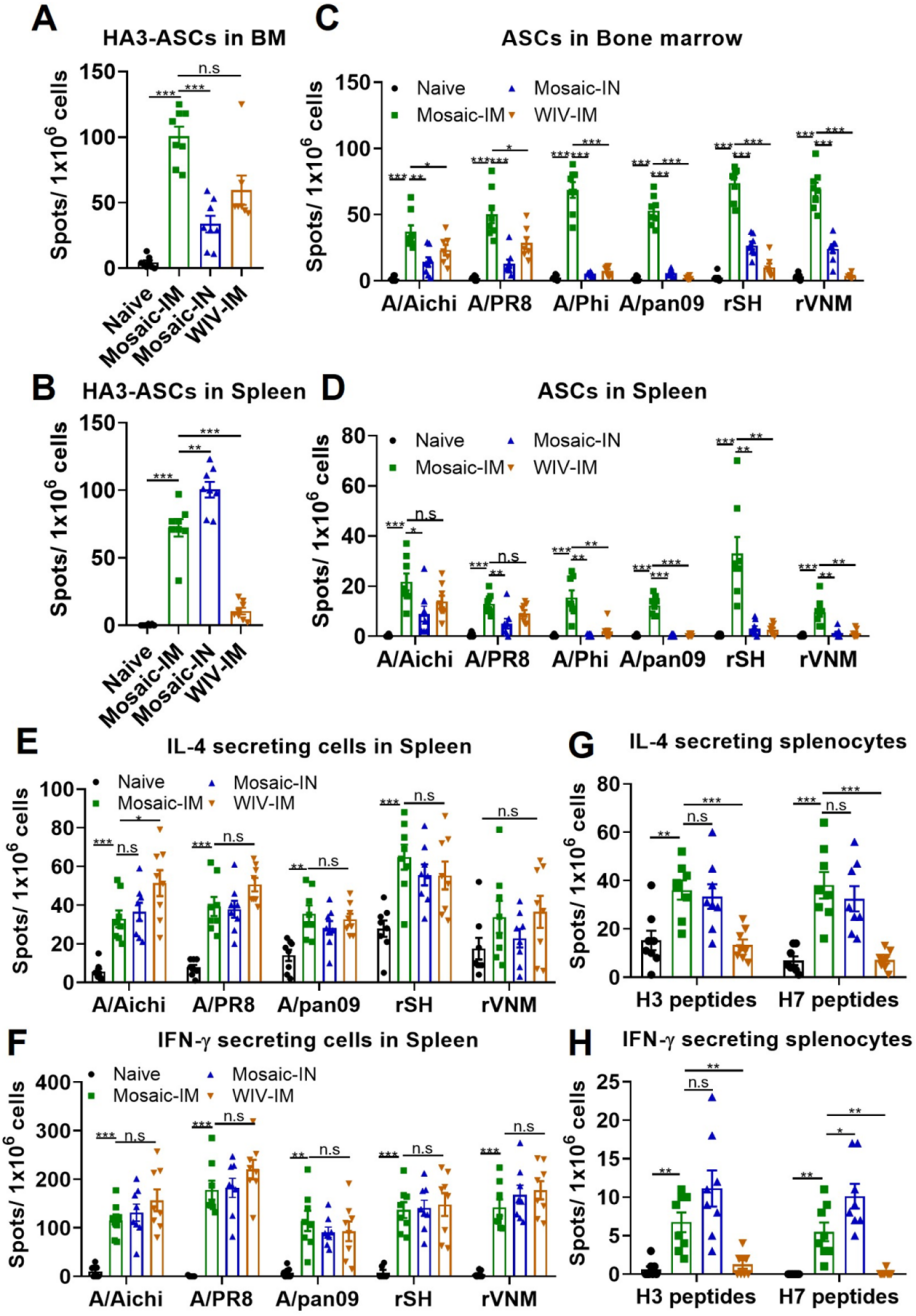
Mosaic inverted HA EV vaccines induced broadly systemic cellular immunity against different influenza strains. Mouse spleens and bone marrows were collected 4 weeks post-boost vaccination to determine the cellular immune responses in the systemic compartment. (A) and (B) HA3-specific IgG-secreting cells (ASCs) in bone marrows and spleens. (C) and (D) A/Aichi, A/PR8, A/Phi, A/pan09, rSH, and rVNM virus-specific ASCs in bone marrows and spleens. (E) and (F) IL-4- and IFN-γ-secreting cells in spleens after stimulation with A/Aichi, A/PR8, A/pan09, rSH or rVNM virus. (G) and (H) IL-4- and IFN-γ-secreting splenocytes after stimulation with H3 or H7 peptide pools. *P* < 0.05 (*), *P* < 0.01 (**), *P* < 0.001 (***), *P* > 0.05 (n.s.).

### Intranasal Delivery of EV-Based Vaccines Induced KLRG1^+^ Memory CD8 T Cells

The KLRG1^+^ CD8 memory population represents a long-lived effector cell (LLEC) subset that combines the features of short-lived effectors and long-lived memory, providing strong protection during acute viral rechallenge.^39^ Splenocytes collected from Mosaic-IM immunized mice showed similar KLRG1 and CD127 expressions to those of the T cells separated from naïve mice. In contrast, Mosaic-IN vaccination significantly increased the expression of KLRG1 and CD127 double-positive CD8 and CD4 T cell populations in spleens 4 weeks post-boosting vaccinations (Figure S4A and B). Further analysis of CD8 subsets revealed a significant increase in KLRG1^+^ CD8 Tcm (central memory, CD62L^+^CD44^+^) and CD8 Tem (effector memory, CD62L^–^CD44^+^) cell populations after intranasal immunization, regardless of CD127 expression (Figure 4A and B). The KLRG1 expression was only slightly increased on CD4 Tcm after Mosaic-IN vaccination, compared with Mosaic-IM (Figure S5A and B), suggesting that Mosaic-IN vaccinations uniquely regulate the KLRG1^+^ CD8 T cells. High-dimensional flow cytometry was used to visualize the expression of KLRG1 and CD127 on CD8 subsets in Figure 4C. Mosaic-IN uniquely stimulated KLRG1^+^ CD8 Tcm (blue and red circles) and KLRG1^+^CD127^–^ CD8 Tem (pink circle) populations, instead of KLRG1^–^CD127^+^ CD8 Tcm or Tem populations (purple and black circles), when compared to Mosaic-IM.

**Figure 4:**
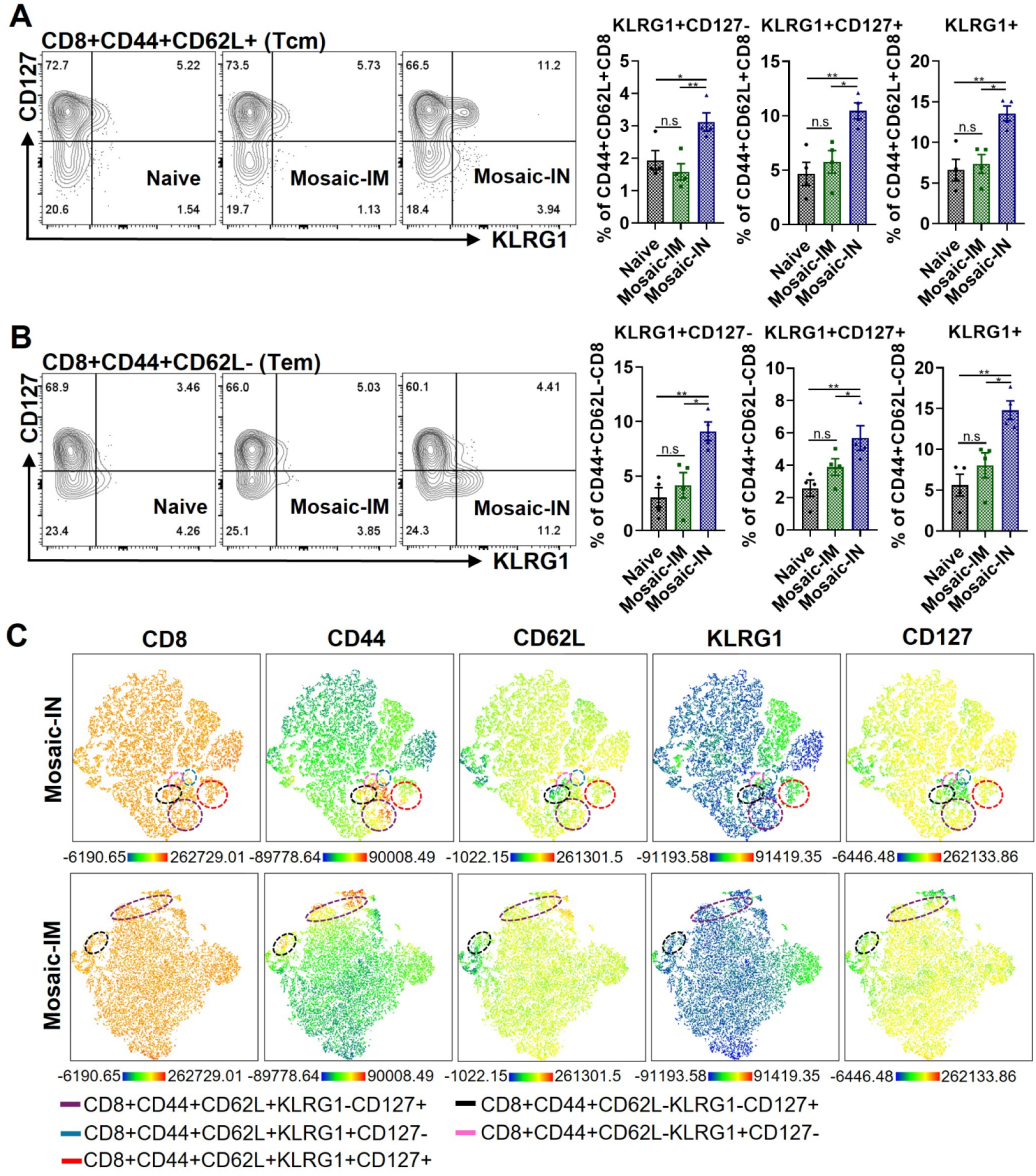
Induction of KLRG1^+^memory CD8 T cells after intranasal vaccination. Mouse splenocytes were stained to determine the expression of KLRG1 and CD127 in different CD8 T cell populations. (A) and (B) The expression of KLRG1 and CD127 on CD8^+^ Tcm and CD8^+^ Tem populations. (C) High-dimensional flow cytometry of CD8 T cell subsets in the vaccinated splenocytes. The distribution of surface markers is displayed in a t-SNE map. Relative marker expression is visualized by the color tone (from blue to orange). Different CD8 T cell subsets were marked with colored dashed circles. *P* < 0.05 (*), *P* < 0.01 (**), *P* < 0.001 (***), *P* > 0.05 (n.s.).

### Intranasal Immunization with Mosaic HA EVs Promoted Strong Mucosal Immune Responses

Nasal washes and bronchoalveolar lavage fluids (BALFs) were collected 4 weeks post-boosting immunization. Compared to other groups, Mosaic-IN enhanced homologous, heterologous, and heterosubtypic virus-specific IgA, as well as HA3 and HA1 stalk-specific IgA, in nasal washes (Figure 5A and B). Similarly, higher levels of virus and HA stalk-specific IgA were observed in BALFs from Mosaic-IN immunized mice (Figure 5C and D). Additionally, Mosaic-IM, Mosaic-IN, or WIV-IM immunization generated comparable levels of antigen-specific IgG in BALFs (Figure 5E). At the same time, Mosaic-IN outperformed others in inducing HA stalk-specific IgG in BALFs (Figure 5F). Consistent with mucosal antibody responses, BALFs from Mosaic-IN-immunized mice showed elevated CD4 and CD8 T cells with higher CD44 expression (Figure 5G). These results manifest that EV-based influenza vaccines effectively target the respiratory system, eliciting potent mucosal IgA antibody and cellular immune responses in the respiratory tract. Thus, EVs present a promising strategy for mucosal vaccines.

**Figure 5:**
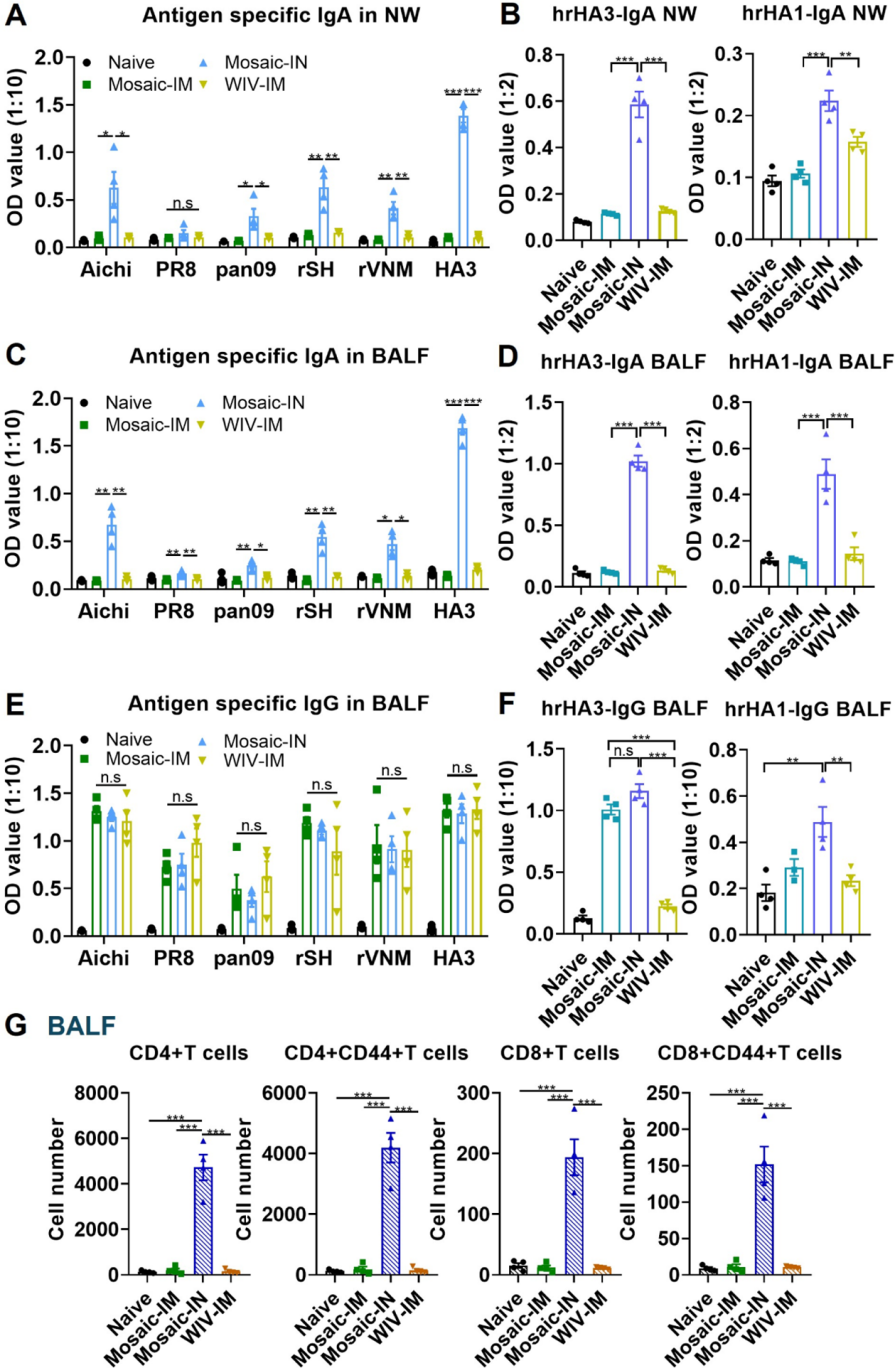
Intranasal immunization of mosaic HA-EVs induced enhanced mucosal immune responses in the respiratory region. Mouse nasal washes and BALFs were collected 4 weeks post-boost vaccination to determine localized antibody and T cell immune responses. The virus- (A) and HA stalk- (B) specific IgA in nasal washes. The virus- and HA stalk-specific IgA ((C) and (D)) and IgG ((E) and (F)) in BALFs. (G) CD4 and CD8 T cell populations in BALFs. *P* < 0.05 (*), *P* < 0.01 (**), *P* < 0.001 (***), *P* > 0.05 (n.s.).

### Mosaic EV Vaccines Protected against Reassortant H5N1 and H7N9 Influenza Virus Infection

Mice were challenged with 3× LD50 of heterosubtypic influenza viruses 4 weeks post-boost vaccinations (Figure 6A). Body weights were monitored for 14 days after infection. In the naive control group, mice continued to lose weight after rSH (H7N9) infection, reaching the humane end point (>20% weight loss) by days 7–8 (Figure 6B). In the Mosaic-IM group, mice exhibited an average body weight loss of 19% by day 7 following rSH infection. They began to recover from day 8. Mice that received Mosaic-IN or WIV-IM experienced less body weight loss (average of 10%) than the Mosaic-IM group and were completely protected against rSH infection (Figure 6C). Similarly, mice immunized with Mosaic-IN and WIV-IM lost 10% of their body weight on day 7 after rVNM (H5N1) infection before recovering (Figure 6D). Compared to the Mosaic-IM group, Mosaic-IN and WIV-IM immunizations fully protected against rVNM (H5N1) infection (Figure 6E). These results demonstrate that EV-based mosaic influenza vaccines elicit protective efficacy against heterosubtypic infections through mucosal immunization, reinforcing EVs as an effective mucosal-targeted vaccine strategy.

**Figure 6:**
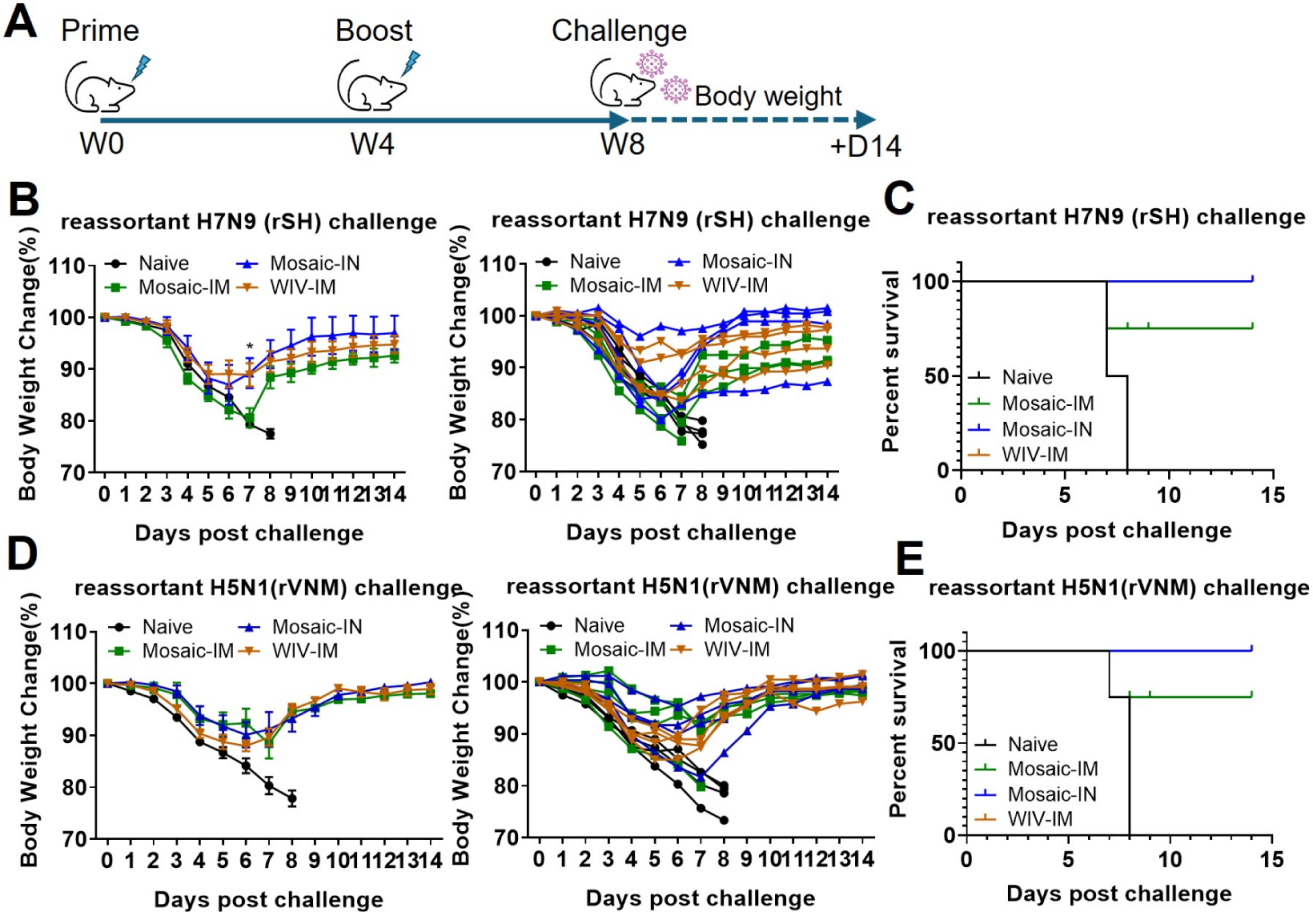
Mucosal vaccination with mosaic HA-EVs provided complete cross-protection against infection with reassortant H5 and H7 viruses in mice. (A) The timeline of the mouse challenge study. The immunized mice were challenged with 3× LD50 of reassortant influenza viruses 4 weeks post-boosting immunization. Mouse body weights were monitored for 14 days post-rSH (B) and rVNM (D) infection, respectively. (C) and (E) The mouse survival rates after rSH and rVNM infection. Kaplan–Meier survival curves are shown for four vaccine groups (*n* = 4 per group). Although two groups showed 100% survival and Naive 0% survival, the global log-rank (Mantel-Cox) test across all groups did not reach statistical significance (*P* = 0.0843). *P* < 0.05 (*).

## Discussion

Seasonal influenza vaccines have been previously shown to confer partial protection against H5N1 in mice and ferrets by inducting cross-reactive antibodies, including HA- and NA-reactive antibodies.^40–42^ Whole-inactivated virus (WIV) vaccines induce cross-protective effects by eliciting CD8^+^ T cell responses targeting conserved internal viral proteins.^43,44^ The reassortant H5N1 and H7N9 challenge viruses used in this study were generated by reverse genetics on a PR8 (A/Puerto Rico/8/34) backbone, containing six internal gene segments from PR8 and the HA and NA segments from avian influenza viruses A/Vietnam/1203/2004 (H5N1) and A/Shanghai/2/2013 (H7N9). Therefore, immunization with PR8 and Aichi WIVs likely induced immune responses targeting these conserved internal proteins, in addition to generating cross-reactive HA and NA antibodies. Our observations indicate that inactivated virus vaccines failed to induce heterosubtypic HA-specific antibodies or HA peptide-specific cytokine-secreting cells. However, they promoted antibody responses and IL-4/IFN-γ-secreting splenocytes against heterologous or heterosubtypic influenza viruses. This suggests that other conserved influenza antigens in the inactivated vaccine formulation might play critical roles in eliciting cross-reactive immune responses. Therefore, incorporating conserved antigens in EV-based mosaic inverted HA vaccines might further boost cross-reactive immunity and protection. To achieve this goal, signature proteins on EV surfaces can facilitate the display of external antigens.^45,46^ Meanwhile, genetic engineering approaches may significantly increase EV yield, thereby enabling scalable production of EV-based vaccines. Additionally, a variety of innovative strategies have been developed to load mRNA into EVs for immunotherapeutic applications.^47–50^ These studies strongly support the potential to generate antigen mRNA-incorporated EV-based mosaic vaccines, highlighting the promise of mRNA-integrated EV platforms for broad applications in vaccines and treatments against inflammatory or infectious diseases.

The mosaic inverted HA EV vaccine displayed HA stalks through inverted conjugations, partially shielding the immunodominant HA head while preserving and exposing the HA stalk domain, which is known as a target of broadly neutralizing antibodies.^25,51^ The intensive presentation of multiple influenza HA stalks on a single nanoparticle significantly increases the likelihood of generating antibodies against shared conserved regions. Vaccinations with the mosaic HA-EVs induced strong and balanced HA1/HA3-stalks specific IgG1 and IgG2a antibodies, suggesting a robust immune response against conserved sites shared among HA stalks on the EV surface. Although fewer studies have directly identified HA-stalk-specific neutralizing antibodies targeting virus particles,^25^ HA stalk-specific IgG2a antibodies showed a higher affinity for several Fcγ receptors and played a pivotal role in protection by inducing FcγR-mediated ADCC and ADCP.^52–55^ In addition, trimeric HA-receptor conjugation on the EV surface requires only one HA subunit/RBD engagement, which partially exposes the HA head epitopes. The conserved receptor-binding site (RBS) or lateral patch epitopes on the HA head are also the targets of broadly neutralizing antibodies.^56,57^ Inducing and identifying conserved HA stalk and HA head-specific neutralizing antibodies is important in developing a universal influenza vaccine. Further studies are needed to characterize the antibodies induced by the mosaic inverted HA-EV vaccine, including the recognition sites, cross-reactivity, and neutralizing ability. Investigating the mosaic inverted HA-EVs that induce antibody-secreting B cells will facilitate the discovery of innovative broadly neutralizing antibodies.

Although intramuscular injection of the mosaic inverted HA EVs showed advantages in inducing broadly cross-reactive antibodies and the antibody-secreting B cells, intranasal delivery of the mosaic EV vaccine elicited additionally strong and broad virus-specific mucosal immune responses in the respiratory tract. Mucosal IgA plays a crucial role in protecting against initial respiratory virus infections.^58^ Broadly cross-reactive HA-specific polymeric secretory IgA (pSIgA), even in the absence of neutralizing activity, has demonstrated cross-protective immunity against influenza viruses.^59^ Despite HA stalk-specific IgA being understudied, it has been shown to exhibit antiviral functions.^60^ In this study, intranasal delivery of mosaic inverted HA EV vaccines promoted HA stalk and various human influenza virus-specific IgA in nasal washes and BALFs. The induction of these IgA in mucosal tissues is expected to enhance protection against influenza viruses at mucosal sites. Notably, the induced IgA may target shared conserved antigenic sites on both HA stalks and HA heads, highlighting the need for further investigation into their protective immunity.

Besides the elevated virus-specific cytokine-secreting cells induced by the mosaic HA-EV vaccines, there were increased KLRG1^+^ CD8 memory T cells in the spleen, detectable only after intranasal vaccination. It has been reported that KLRG1^+^ CD8 T cells can survive for months after acute infection, retain effector function and protective immunity, and have the flexibility to enter tissues and establish residence.^61^ In addition, we found that mice immunized with EV influenza vaccines exhibited significant CD8 T_RM_ populations in the lung.^15^ The enhanced KLRG1^+^ CD8 memory populations may contribute to the enrichment of CD8 T_RM_ in the lung after intranasal vaccination. Further studies are needed to demonstrate the relationship between splenic KLRG1^+^ CD8 memory T cells and lung CD8 T_RM_ induced by intranasal immunization.

Vaccination with the mosaic inverted HA EV vaccine strongly stimulated cross-reactive antibodies and cellular immune responses against various human influenza strains, and especially provided complete protection against the heterosubtypic reassortant H5N1 and H7N9 infections after intranasal vaccination. This study suggested that appropriately displayed HA-stalks from low-pathogenic avian influenza (LPAI) strains by EVs might induce cross-immunity against highly pathogenic avian influenza (HPAI) strains. The devastating impact of the HPAI outbreak on the poultry industry is widely recognized. However, the threat posed by LPAI viruses, which can potentially evolve into highly pathogenic strains upon transmission to domestic poultry, has been underestimated.^62–65^ Acquiring immunity against HA conserved domains from a variety of human and avian strains may help prepare for the sudden emergence of HPAI and mutated LPAI. Considering the safety profiles of EVs, the innovative mosaic formulation based on EVs is a promising platform for combining multiple influenza antigens to stimulate and establish effective, cross-reactive immunity in the respiratory system. Well-established cell culture systems and bioreactor-based manufacturing will facilitate the roadmap for extensive animal studies and the future human translation of engineered EVs for vaccines and therapeutics.

## Conclusions

In conclusion, we developed a mosaic influenza vaccine platform based on extracellular vesicles (EVs) that display multiple HAs from both human and avian influenza viruses on their surfaces in an inverted orientation. Immunization with these mosaic HA-EVs, through either intramuscular or intranasal routes, elicited strong cross-reactive HA-stalk and virus-specific IgG responses, as well as robust cellular immune responses in systemic compartments. Notably, intranasal administration significantly enhanced HA-stalk- and virus-specific IgA responses in the respiratory tract, including nasal washes and BALFs, and increased the population of KLRG1^+^ CD8 T cells in the spleen. Intranasal immunization with mosaic HA-EV vaccines conferred complete protection against lethal heterosubtypic challenges with H7N9 and H5N1 reassortants. Collectively, these results highlight EVs as a promising platform for mucosal vaccine delivery and suggest their potential in developing universal HA stalk-based influenza vaccines.

## Methods

### Ethical Statement

All animal studies were performed according to the Guide for the Care and Use of Laboratory Animals of the National Institutes of Health. The mice used in this work were maintained at the Georgia State University animal facility, and the studies were approved by the Georgia State University Institutional Animal Care and Use Committee under protocol No. A20027.

### Plasmids, Recombinant Proteins, and Viruses

The full-length HA plasmids including A/mallard/Sweden/86/2003 (H12N5) (NR-29004), A/Eurasian wigeon/Netherlands/4/2005 (H9N2) (NR-29001), A/black headed gull/Sweden/1/1999 (H13N6) (NR-29005), A/mallard/Netherlands/1/1999 (H4N6) (NR-28996) and A/mallard/Sweden/51/2002 (H10N2) (NR-29002) were obtained from BEI Resources under the NIH research reagent sharing program. The HA constructs without the transmembrane domain were fused with GCN4 and a 6xHis-tag at the C-terminus and cloned into the pFastBac-1 vector. The recombinant bacmids and baculoviruses (rBVs) were generated and produced using the Bac-to-Bac Baculovirus Expression System purchased from Thermo Fisher Scientific. The recombinant His-tagged HA1, HA3, HA12, HA9, HA4, and HA10 proteins were expressed in rBV-infected Sf9 insect cells and purified using HisPur Ni-NTA Resin (Thermo Scientific). A/Aichi/2/1968 (A/Aichi, H3N2), A/Philippines/2/1982 (A/Phi, H3N2), A/Puerto Rico/8/1934 (A/PR8, H1N1), A/California/07/2009­(A/pan09, H1N1), reassortant A/Shanghai/2/2013 (rSH, H7N9), and reassortant A/Vietnam/1203/2004 (rVNM, H5N1) influenza viruses were amplified in embryonated chicken eggs and purified by sucrose gradient ultracentrifugation. The rVNM and rSH viruses used in this study were generated from an A/PR8 background by reverse genetics, containing the six internal gene segments from A/PR8 and the HA and NA from H5N1 or H7N9 avian influenza viruses.^52,66^ The influenza viruses were handled in a biosafety level 2 laboratory.

### Generation and Characterization of EVs-Based Mosaic Influenza Vaccine

The 293T cell line (ATCC, CRL-3216) was cultured for EV production as previously described.^15^ Briefly, the supernatant was collected after 3 days of culture and centrifuged sequentially at 500x *g* and 5000x *g* for 10 and 30 min, respectively, to remove all cell debris. The supernatant was then subjected to ultracentrifugation at 27,800x rpm for 2 h (TH-641, Sorvall WX+ Ultracentrifuge Series).^67^ The pelleted EVs were washed and ultracentrifuged with Dulbecco’s phosphate-buffered saline (DPBS) to completely remove the culture medium, then dissolved in DPBS at 4 °C overnight. The solubilized EVs were further centrifuged at 20,000x *g* for 20 min to remove large vesicles, and the remaining EVs in the supernatant were used for vaccine manufacturing. The trimeric HA proteins, including HA1, HA9, HA12, HA3, HA4, and HA10, were generated and purified as previously described.^68^ The concentrations of EVs and HA proteins were determined by the Micro BCA Protein Assay Kit (Thermo Scientific). A similar strategy was performed to produce Group 1 and Group 2 mosaic influenza HA stalk vaccines containing HA1/HA9/HA12 or HA3/HA4/HA10 separately. To generate a mosaic vaccine containing Group 1 influenza virus HAs, the HA1/HA9/HA12 proteins were incubated with purified EVs at a 1:1:1:1 weight ratio at 4 °C overnight, then washed twice with DPBS to remove unbound HA proteins. The pellets were dissolved in DPBS at 4 °C overnight, and the undissolved large vesicles were removed by centrifugation. The size distribution of EVs was measured by Dynamic Light Scattering (DLS) analysis (Malvern Zetasizer). The TEM imaging was performed at the Emory University Robert P. Apkarian Integrated Electron Microscopy Core Facility (RRID: SCR_023537). The total amount of HA proteins on EVs was determined by SDS-PAGE and Coomassie blue staining. The HA-EV vaccine was analyzed by Western blot using antibodies against Alix (Cat. No. 634501, Biolegend), TSG101 (Cat. No. 934301, Biolegend), CD81 (Cat. No. 27855-1-AP, Proteintech), and β-actin (Cat. No. 20536-1-AP, Proteintech).

### Animal Immunization and Infection

The female BALB/c mice (6-to-8-week-old) were intramuscularly or intranasally immunized with two doses of EV-based mosaic HA influenza vaccines containing 6 μg of total HAs from the mixed Group 1 and Group 2 mosaic vaccines at four-week intervals (designated as Mosaic-IM and Mosaic-IN). Groups of naïve mice and mice intramuscularly immunized with the bivalent whole inactivated A/Aichi and A/PR8 viruses (WIV-IM, containing 1 μg of each HA1 and HA3) were included as controls. At week four post-boosting vaccination, mice were intranasally infected with 3× LD50 of rSH (H7N9) or rVNM (H5N1).^52^ The condition of the mice was closely monitored, and the body weights were recorded for 14 days after infection. Mice were euthanized when body weight dropped by more than 20% of the initial value.

### ELISA for Antibody Immune Responses

Blood was collected from mice 3 weeks after each immunization, and the sera were analyzed for antigen-specific IgG and IgG subtypes. Mice were euthanized 4 weeks post-boosting vaccinations. Nasal washes and BALFs were collected to evaluate the antigen-specific IgG and IgA antibodies. Briefly, 4 μg/mL of different inactivated influenza viruses, purified HA, and HA-stalk proteins were used to coat the plate at 4 °C overnight. The plates were washed and blocked in PBST containing 2% BSA at 37 °C for 1 h, then incubated with the serially diluted sera. Two hours after incubation, the plates were washed and incubated with the diluted HRP-conjugated goat antimouse IgG, IgG1, IgG2a, or IgA secondary antibodies (SouthernBiotech) for 1 h. The TMB substrate solution (Thermo Scientific) was used for HRP detection, and the reaction was stopped by adding an equal volume of 1 M H_2_SO_4_. The plates were read at 450 nm by a BioTek Microplate Reader.

To assess CR9114 (Cell Sciences, DVV03807A) binding to mosaic HA-EVs, various concentrations of mosaic HA-EVs and control EVs were coated onto ELISA plates and incubated with 4 μg/mL of CR9114, followed by detection with a HRP-conjugated goat anti-human IgG secondary antibody (Thermo Scientific). Additionally, 6 μg/mL of mosaic HA-EVs were coated on plates and incubated with different concentrations of CR9114. To evaluate binding inhibition, sera from immunized mice were diluted 1:100 and mixed with CR9114 prior to incubation.

### BMDC Stimulation

Bone marrow cells were isolated and seeded at 1 × 10^6^ cells/mL in complete RPMI1640 medium containing glutamine, penicillin/streptomycin, 2-mercaptoethanol, HEPES, nonessential amino acid, sodium pyruvate, 10% FBS, and 20 ng/mL GM-CSF. Cells were cultured at 37 °C, and half the medium was refreshed with GM-CSF-supplemented medium on day 2. Nonadherent and loosely adherent cells were pelleted and resuspended in fresh medium on day 3, followed by 3 more days of culture. On day 6, cells were harvested and stimulated with 5 μg/mL mosaic HA-EVs or MPLA. After 18 h, cells and supernatants were collected. Cells were stained with anti-mouse CD11c-APC, CD40-PE, CD86-PE-Cy7, CD16/32 antibodies, and Zombie NIR dye-APC-Cy7, and then analyzed on a BD CytoFLEX Flow Cytometer. TNF-α and IL-12/p70 levels in supernatants were measured by ELISA. Captured antibodies (4 μg/mL) were coated on plates overnight at 4 °C. Supernatants (50 μL) were added and incubated at 37 °C for 2 h, followed by biotin-conjugated detection antibodies and HRP-conjugated streptavidin (BioLegend). Serial dilutions of TNF-α and IL-12/p70 standards were used to generate standard curves.

### Neutralization and Hemagglutination Inhibition (HAI) Assay

Madin-Darby canine kidney (MDCK) cells (5 × 10^3^ cells in 100 μL per well) were seeded into 96-well plates for the neutralization assay. Immune sera were serially diluted and incubated with an equal volume of influenza virus (100 TCID_50_) for 1 h. The serum-virus mixtures were then added to MDCK-seeded 96-well plates and incubated at 37 °C for 3–5 days to observe virus-induced cytopathic effects. Data were analyzed using GraphPad Prism 8.0.1. Nonlinear regression with dose–response inhibition was used to calculate IC_50_ values.

A modified HAI assay was performed as previously described.^17,69^ Briefly, boost sera were treated with the receptor-destroying enzyme (RDE, Denka Seiken) at a 1:3 ratio and incubated at 37 °C overnight, followed by heat inactivation at 56 °C for 30 min. DPBS was then added to each sample to achieve a 1:10 dilution. The treated sera were serially 2-fold diluted and incubated with 4 HA units/25 μL of Aichi or PR8 virus for 30 min. 50 μL of 1.0% horse red blood cells (Innovative Research Inc.) were added, and agglutination was observed after 1 h. The HAI titers were defined as the highest serum dilution that inhibited hemagglutination.

### ELISPOT for Cytokine-Secreting Cells

Mice spleens and bone marrows were collected one month post-boosting immunization for analysis of IL-4- and IFN-γ-secreting cells.^17,70^ The MultiScreen filter plates (Millipore) were coated with 4 μg/mL of anti-IL-4 and anti-IFN-γ capture antibodies at 4 °C 1 day before cell seeding. The plates were washed with DPBS and incubated with complete RPMI1640 medium for 2 h. One million isolated splenocytes were added to each well in the presence of stimulation with different purified viruses, including A/Aichi/2/1968 (H3N2), A/Philippines/2/1982 (H3N2), A/Puerto Rico/8/1934 (H1N1), A/California/07/2009 (H1N1), rSH (H7N9), or rVNM (H5N1). The splenocytes were stimulated with HA peptide pools from A/Anhui/1/2013 (H7N9) (NR-44011) and A/Wisconsin/67e5/2005 (H3N2) (NR-9472), which were obtained from BEI Resources. After 3 days of culture, the plates were washed with PBST, and cytokines were measured using biotinylated anti-IL-4 or anti-IFN-γ antibodies and streptavidin-HRP secondary antibody (BioLegend). To determine the antibody-secreting cells (ASCs) in spleens and bone marrows, the plates were coated with 4 μg/mL of purified virus or HA protein at 4 °C overnight. After blocking, one million single-cell suspensions were added and cultured at 37 °C for 18 h. The goat anti-mouse IgG-HRP was used to detect ASCs. The colonies were stained with KPL True Blue substrate (SeraCare) and recorded by Bioreader-6000-E (BIOSYSTEM).

### Flow Cytometry

Mice spleens were collected and processed into single-cell suspensions one month post-boosting immunization. The fresh splenocytes were stained with anti-mouse CD45-PE, CD4-PerCP, CD8-FITC, CD44-BV421, CD62L-APC-Cy7, CD127-APC, KLRG1-BV510, and CD16/32 antibodies (BioLegend) in DPBS containing 2% FBS (FACS buffer) for 20 min on ice. The cells were washed and resuspended in FACS buffer, then analyzed on a BD LSRFortessa Cell Analyzer. Lymphocytes in BALFs were separated and stained with anti-mouse CD45-PE, CD4-PerCP, CD8-FITC, CD44-BV421, CD16/32 antibodies, and Zombie NIR dye-APC-Cy7 (BioLegend). The flow cytometry data were analyzed using FlowJo, and t-SNE plots were generated to visualize the CD8 populations in the spleens.

### Statistical Analysis

Statistical analysis of the data set containing three or more groups was performed in GraphPad Prism 8.0.1. The data set’s normality was evaluated using the Shapiro-Wilk test. The Brown-Forsythe test was used to determine whether standard deviations (SDs) differed significantly. Ordinary one-way ANOVA and Tukey’s multiple comparisons tests were applied when SDs had no difference. With the SDs showing significant differences, Brown-Forsythe and Welch ANOVA tests followed by Games–Howell’s multiple comparison test were used. For the comparison of data with non-normal distribution, the Kruskal–Wallis and Dunn’s multiple comparisons tests were used. To compare two groups of data with normal distribution, p-values were calculated by the F-test and two-tailed Student’s *t*-test. The Mann–Whitney U test was used to compare two groups with non-normally distributed data. The data were represented by means with standard error of the mean (SEM). A p-value <0.05 was statistically significant. *p* < 0.05 (*), *p* < 0.01 (**), *p* < 0.001 (***), *p* > 0.05 (n.s.). Survival curves were plotted using the Kaplan–Meier method and compared using the log-rank (Mantel–Cox) test in GraphPad Prism. A *p*-value <0.05 was considered statistically significant.

## Supplementary Material



## Notes

Characterization of purified EVs with and without CD81; ELISA OD values at different serum dilutions; additional analysis of KLRG-1 expression in spleen T cell populations (PDF)

## References

[1] Calzas C., Chevalier C. (2019). Innovative Mucosal Vaccine Formulations against Influenza
a Virus Infections. Front. Immunol..

[2] Lavelle E. C., Ward R. W. (2022). Mucosal Vaccines - Fortifying the
Frontiers (Aug, 2021,
10.1038/S41577–021–00583–2). Nat. Rev. Immunol..

[3] Dhama K., Dhawan M., Tiwari R., Emran T. B., Mitra S., Rabaan A. A., Alhumaid S., Alawi Z. A., Al Mutair A. (2022). Covid-19 Intranasal
Vaccines: Current Progress, Advantages, Prospects, and Challenges. Hum. Vaccines Immunother..

[4] Eshaghi B., Schudel A., Sadeghi I., Chen Z. Q., Lee A. H., Kanelli M., Tierney F., Han J., Ingalls B., Francis D. M., Li G., von Andrian U., Langer R., Jaklenec A. (2024). The Role of Engineered Materials
in Mucosal Vaccination Strategies. Nat. Rev.
Mater..

[5] Kiyono H., Ernst P. B. (2025). Nasal Vaccines for Respiratory Infections. Nature.

[6] Fausther-Bovendo H., Kobinger G. P. (2014). Pre-Existing Immunity
against Ad Vectors: Humoral,
Cellular, and Innate Response, What’s Important?. Hum. Vaccines Immunother..

[7] An D., Li K., Rowe D. K., Diaz M. C. H., Griffin E. F., Beavis A. C., Johnson S. K., Padykula I., Jones C. A., Briggs K. (2021). Protection
of K18-hACE2 mice and ferrets against SARS-CoV-2 challenge
by a single-dose mucosal immunization with a parainfluenza virus 5–based
COVID-19 vaccine. Sci. Adv..

[8] Spearman P., Jin H., Knopp K., Xiao P., Gingerich M. C., Kidd J., Singh K., Tellier M., Radziewicz H., Wu S. (2023). Intranasal Parainfluenza
Virus Type 5 (Piv5)-Vectored
Rsv Vaccine Is Safe and Immunogenic in Healthy Adults in a Phase 1
Clinical Study. Sci. Adv..

[9] Kaiser J. A., Liu X., Luongo C., Matsuoka Y., Santos C., Yang L., Herbert R., Castens A., Dorward D. W., Johnson R. F., Park H. S., Afroz S., Munir S., Le Nouen C., Buchholz U. J. (2023). Intranasal Murine
Pneumonia Virus-Vectored Sars-Cov-2
Vaccine Induces Mucosal and Serum Antibodies in Macaques. iScience.

[10] Kaiser J. A., Nelson C. E., Liu X. Q., Park H. S., Matsuoka Y., Luongo C., Santos C., Ahlers L. R. H., Herbert R., Moore I. N. (2024). Mucosal Prime-Boost
Immunization with Live
Murine Pneumonia Virus-Vectored Sars-Cov-2 Vaccine Is Protective in
Macaques. Nat. Commun..

[11] Zhang B., Sim W. K., Shen T.-L., Lim S. K. (2024). Engineered
Evs with
Pathogen Proteins: Promising Vaccine Alternatives to Lnp-Mrna Vaccines. J. Biomed. Sci..

[12] Santos P., Almeida F. (2021). Exosome-Based Vaccines:
History, Current State, and
Clinical Trials. Front. Immunol..

[13] Cocucci E., Meldolesi J. (2015). Ectosomes
and Exosomes: Shedding the Confusion between
Extracellular Vesicles. Trends Cell Biol..

[14] Meldolesi J. (2018). Exosomes and
Ectosomes in Intercellular Communication. Curr.
Biol..

[15] Zhu W., Dong C., Wei L., Kim J. K., Wang B.-Z. (2025). Inverted
Ha-Ev Immunization Elicits Stalk-Specific Influenza Immunity and Cross-Protection
in Mice. Mol. Ther..

[16] Dong C., Wei L., Zhu W., Kim J. K., Wang Y., Omotara P., Arsana A., Wang B. Z. (2025). Mature Dendritic Cell-Derived Extracellular
Vesicles Are Potent Mucosal Adjuvants for Influenza Hemagglutinin
Vaccines. ACS Nano.

[17] Zhu W., Pewin W., Wang C., Luo Y., Gonzalez G. X., Mohan T., Prausnitz M. R., Wang B. Z. (2017). A Boosting Skin
Vaccination with Dissolving Microneedle Patch Encapsulating M2e Vaccine
Broadens the Protective Efficacy of Conventional Influenza Vaccines. J. Controlled Release.

[18] Zhu W., Park J., Pho T., Wei L., Dong C., Kim J., Ma Y., Champion J. A., Wang B.-Z. (2023). Iscoms/Mpla-Adjuvanted
Sdad Protein Nanoparticles Induce Improved Mucosal Immune Responses
and Cross-Protection in Mice. Small.

[19] Dong C., Zhu W., Wei L., Kim J. K., Ma Y., Kang S.-M., Wang B.-Z. (2024). Enhancing Cross-Protection against
Influenza by Heterologous
Sequential Immunization with Mrna Lnp and Protein Nanoparticle Vaccines. Nat. Commun..

[20] Zhu W. D., Wei L., Dong C. H., Wang Y., Kim J., Ma Y., Gonzalez G. X., Wang B. Z. (2022). Cgamp-Adjuvanted Multivalent Influenza
Mrna Vaccines Induce Broadly Protective Immunity through Cutaneous
Vaccination in Mice. Mol. Ther. -Nucleic Acids.

[21] Xia Y., Zhang J., Liu G., Wolfram J. (2024). Immunogenicity of Extracellular
Vesicles. Adv. Mater..

[22] Steel J., Lowen A. C., Wang T. T., Yondola M., Gao Q., Haye K., Garcia-Sastre A., Palese P. (2010). Influenza Virus Vaccine
Based on the Conserved Hemagglutinin Stalk Domain. mBio.

[23] Isakova-Sivak I., Rudenko L. (2022). The Future of Haemagglutinin
Stalk-Based Universal
Influenza Vaccines. Lancet Infect. Dis..

[24] Krammer F. (2019). The Human
Antibody Response to Influenza a Virus Infection and Vaccination. Nat. Rev. Immunol..

[25] Guthmiller J. J., Han J., Utset H. A., Li L., Lan L. Y., Henry C., Stamper C. T., McMahon M., O’Dell G., Fernandez-Quintero M.
L., Freyn A. W., Amanat F., Stovicek O., Gentles L., Richey S. T., de la
Pena A. T., Rosado V., Dugan H. L., Zheng N. Y., Tepora M. E., Bitar D. J., Changrob S., Strohmeier S., Huang M., Garcia-Sastre A., Liedl K. R., Bloom J. D., Nachbagauer R., Palese P., Krammer F., Coughlan L., Ward A. B., Wilson P. C. (2022). Broadly Neutralizing Antibodies Target
a Haemagglutinin Anchor Epitope. Nature.

[26] Wrammert J., Koutsonanos D., Li G. M., Edupuganti S., Sui J., Morrissey M., McCausland M., Skountzou I., Hornig M., Lipkin W. I., Mehta A., Razavi B., Del Rio C., Zheng N. Y., Lee J. H., Huang M., Ali Z., Kaur K., Andrews S., Amara R. R., Wang Y., Das S. R., O’Donnell C. D., Yewdell J. W., Subbarao K., Marasco W. A., Mulligan M. J., Compans R., Ahmed R., Wilson P. C. (2011). Broadly Cross-Reactive Antibodies Dominate the Human
B Cell Response against 2009 Pandemic H1n1 Influenza Virus Infection. J. Exp. Med..

[27] Nachbagauer R., Feser J., Naficy A., Bernstein D. I., Guptill J., Walter E. B., Berlanda-Scorza F., Stadlbauer D., Wilson P. C., Aydillo T., Behzadi M. A., Bhavsar D., Bliss C., Capuano C., Carreno J. M., Chromikova V., Claeys C., Coughlan L., Freyn A. W., Gast C., Javier A., Jiang K., Mariottini C., McMahon M., McNeal M., Solorzano A., Strohmeier S., Sun W., Van der Wielen M., Innis B. L., Garcia-Sastre A., Palese P., Krammer F. (2021). A Chimeric
Hemagglutinin-Based Universal Influenza Virus Vaccine Approach Induces
Broad and Long-Lasting Immunity in a Randomized, Placebo-Controlled
Phase I Trial. Nat. Med..

[28] Folschweiller N., Vanden Abeele C., Chu L., Van Damme P., Garcia-Sastre A., Krammer F., Nachbagauer R., Palese P., Solorzano A., Bi D. (2022). Reactogenicity,
Safety, and Immunogenicity of Chimeric Haemagglutinin Influenza Split-Virion
Vaccines, Adjuvanted with As01 or As03 or Non-Adjuvanted: A Phase
1–2 Randomised Controlled Trial. Lancet
Infect. Dis..

[29] Houser K. V., Chen G. L., Carter C., Crank M. C., Nguyen T. A., Burgos Florez M. C., Berkowitz N. M., Mendoza F., Hendel C. S., Gordon I. J. (2022). Safety and Immunogenicity of a Ferritin Nanoparticle
H2 Influenza Vaccine in Healthy Adults: A Phase 1 Trial. Nat. Med..

[30] Widge A. T., Hofstetter A. R., Houser K. V., Awan S. F., Chen G. L., Burgos Florez M. C., Berkowitz N. M., Mendoza F., Hendel C. S., Holman L. A. (2023). An
Influenza Hemagglutinin Stem Nanoparticle
Vaccine Induces Cross-Group 1 Neutralizing Antibodies in Healthy Adults. Sci. Transl. Med..

[31] Casazza J. P., Hofstetter A. R., Costner P. J. M., Holman L. A., Hendel C. S., Widge A. T., Wu R. L., Whalen W. R., Cunningham J., Arthur A. (2024). Phase 1 Dose-Escalation
Trial Evaluating a Group 2
Influenza Hemagglutinin Stabilized Stem Nanoparticle Vaccine. Npj Vaccines.

[32] Kanekiyo M., Joyce M. G., Gillespie R. A., Gallagher J. R., Andrews S. F., Yassine H. M., Wheatley A. K., Fisher B. E., Ambrozak D. R., Creanga A., Leung K., Yang E. S., Boyoglu-Barnum S., Georgiev I. S., Tsybovsky Y., Prabhakaran M. S., Andersen H., Kong W. P., Baxa U., Zephir K. L., Ledgerwood J. E., Koup R. A., Kwong P. D., Harris A. K., McDermott A. B., Mascola J. R., Graham B. S. (2019). Mosaic
Nanoparticle Display of Diverse Influenza Virus Hemagglutinins Elicits
Broad B Cell Responses. Nat. Immunol..

[33] Liu X., Zhao T., Wang L., Yang Z., Luo C., Li M., Luo H., Sun C., Yan H., Shu Y. (2023). A Mosaic Influenza
Virus-Like Particles Vaccine Provides Broad Humoral and Cellular Immune
Responses against Influenza a Viruses. Npj Vaccines.

[34] Cohen A. A., van Doremalen N., Greaney A. J., Andersen H., Sharma A., Starr T. N., Keeffe J. R., Fan C., Schulz J. E., Gnanapragasam P. N. P. (2022). Mosaic Rbd Nanoparticles Protect against
Challenge by Diverse Sarbecoviruses in Animal Models. Science.

[35] Kaabi N. A., Yang Y. K., Liang Y., Xu K., Zhang X. F., Kang Y., Jin Y. Q., Hou J. W., Zhang J., Yang T. (2023). Safety and Immunogenicity of a Mosaic Vaccine Booster
against Omicron and Other Sars-Cov-2 Variants: A Randomized Phase
2 Trial. Signal Transduction Targeted Ther..

[36] Cohen A. A., Keeffe J. R., Schiepers A., Dross S. E., Greaney A. J., Rorick A. V., Gao H., Gnanapragasam P. N. P., Fan C., West A. P. (2024). Mosaic Sarbecovirus Nanoparticles Elicit Cross-Reactive
Responses
in Pre-Vaccinated Animals. Cell.

[37] Beukenhorst A. L., Rogiers R., Rice K. L., Booth G., Haasnoot J., Nkolola J., Ayala J., Wang L., Julg B., Pastini A. K. (2026). Phase
1 and Preclinical Studies Reveal Safety,
Pharmacokinetics, and Efficacy of Intranasal Delivery of the Influenza
Antibody Cr9114. Sci. Transl. Med..

[38] Andreu Z., Yanez-Mo M. (2014). Tetraspanins in Extracellular
Vesicle Formation and
Function. Front. Immunol..

[39] Renkema K. R., Huggins M. A., Borges
da Silva H., Knutson T. P., Henzler C. M., Hamilton S. E. (2020). Klrg1­(+)
Memory Cd8 T Cells Combine Properties of Short-Lived
Effectors and Long-Lived Memory. J. Immunol..

[40] Roos A., Roozendaal R., Theeuwsen J., Riahi S., Vaneman J., Tolboom J., Dekking L., Koudstaal W., Goudsmit J., Radosevic K. (2015). Protection against H5n1 by Multiple
Immunizations with Seasonal Influenza Vaccine in Mice Is Correlated
with H5 Cross-Reactive Antibodies. Vaccine.

[41] Sun X., Subbiah J., Belser J. A., Brock N., Gansebom S., Li Z. N., Jung Y. J., Liu F., Tumpey T. M., Maines T. R. (2025). Effect of Seasonal Influenza Vaccines on Avian
Influenza a­(H5n1) Clade 2.3.4.4b Virus Infection in Ferrets. Emerg. Infect. Dis..

[42] Rockman S., Brown L. E., Barr I. G., Gilbertson B., Lowther S., Kachurin A., Kachurina O., Klippel J., Bodle J., Pearse M., Middleton D. (2013). Neuraminidase-Inhibiting
Antibody Is a Correlate of Cross-Protection against Lethal H5n1 Influenza
Virus in Ferrets Immunized with Seasonal Influenza Vaccine. J. Virol..

[43] Budimir N., de Haan A., Meijerhof T., Gostick E., Price D. A., Huckriede A., Wilschut J. (2013). Heterosubtypic Cross-Protection Induced
by Whole Inactivated Influenza Virus Vaccine in Mice: Influence of
the Route of Vaccine Administration. Influenza
Other Respir. Viruses.

[44] Dong W., Bhide Y., Sicca F., Meijerhof T., Guilfoyle K., Engelhardt O. G., Boon L., de Haan C. A. M., Carnell G., Temperton N. (2018). Cross-Protective Immune
Responses Induced by Sequential Influenza Virus Infection and by Sequential
Vaccination with Inactivated Influenza Vaccines. Front. Immunol..

[45] Kumar M. A., Baba S. K., Sadida H. Q., Marzooqi S. A., Jerobin J., Altemani F. H., Algehainy N., Alanazi M. A., Abou-Samra A. B., Kumar R. (2024). Extracellular
Vesicles as Tools and Targets in Therapy
for Diseases. Signal Transduction Targeted Ther..

[46] Du S., Guan Y., Xie A., Yan Z., Gao S., Li W., Rao L., Chen X., Chen T. (2023). Extracellular Vesicles:
A Rising Star for Therapeutics and Drug Delivery. J. Nanobiotechnol..

[47] You Y., Tian Y., Yang Z., Shi J., Kwak K. J., Tong Y., Estania A. P., Cao J., Hsu W. H., Liu Y., Chiang C. L., Schrank B. R., Huntoon K., Lee D., Li Z., Zhao Y., Zhang H., Gallup T. D., Ha J., Dong S., Li X., Wang Y., Lu W. J., Bahrani E., Lee L. J., Teng L., Jiang W., Lan F., Kim B. Y. S., Lee A. S. (2023). Intradermally Delivered Mrna-Encapsulating
Extracellular Vesicles for Collagen-Replacement Therapy. Nat. Biomed. Eng..

[48] Dong S., Liu X., Bi Y., Wang Y., Antony A., Lee D., Huntoon K., Jeong S., Ma Y., Li X. (2023). Adaptive
Design of Mrna-Loaded Extracellular Vesicles for Targeted
Immunotherapy of Cancer. Nat. Commun..

[49] Malle M. G., Song P., Loffler P. M. G., Kalisi N., Yan Y., Valero J., Vogel S., Kjems J. (2024). Programmable Rna Loading
of Extracellular Vesicles with Toehold-Release Purification. J. Am. Chem. Soc..

[50] Payandeh Z., Tangruksa B., Synnergren J., Heydarkhan-Hagvall S., Nordin J. Z., Andaloussi S. E., Borén J., Wiseman J., Bohlooly-Y M., Lindfors L. (2024). Extracellular
Vesicles Transport Rna between Cells: Unraveling Their Dual Role in
Diagnostics and Therapeutics. Mol. Aspects Med..

[51] Freyn A. W., Han J. L. N., Guthmiller J. J., Bailey M. J., Neu K., Turner H. L., Rosado V. C., Chromikova V., Huang M., Strohmeier S. (2021). Influenza Hemagglutinin-Specific
Iga Fc-Effector Functionality Is Restricted to Stalk Epitopes. Proc. Natl. Acad. Sci. U. S. A..

[52] Deng L., Mohan T., Chang T. Z., Gonzalez G. X., Wang Y., Kwon Y. M., Kang S. M., Compans R. W., Champion J. A., Wang B.-Z. (2018). Double-Layered Protein Nanoparticles
Induce Broad Protection
against Divergent Influenza a Viruses. Nat.
Commun..

[53] Cox F., Kwaks T., Brandenburg B., Koldijk M. H., Klaren V., Smal B., Korse H. J. W. M., Geelen E., Tettero L., Zuijdgeest D. (2016). Ha Antibody-Mediated Fcγriiia Activity
Is Both Dependent on Fcr Engagement and Interactions between Ha and
Sialic Acids. Front. Immunol..

[54] Mullarkey C. E., Bailey M. J., Golubeva D. A., Tan G. S., Nachbagauer R., He W., Novakowski K. E., Bowdish D. M., Miller M. S., Palese P. (2016). Broadly Neutralizing
Hemagglutinin Stalk-Specific Antibodies
Induce Potent Phagocytosis of Immune Complexes by Neutrophils in an
Fc-Dependent Manner. mBio.

[55] DiLillo D. J., Tan G. S., Palese P., Ravetch J. V. (2014). Broadly
Neutralizing
Hemagglutinin Stalk-Specific Antibodies Require Fcgammar Interactions
for Protection against Influenza Virus in Vivo. Nat. Med..

[56] Raymond D. D., Bajic G., Ferdman J., Suphaphiphat P., Settembre E. C., Moody M. A., Schmidt A. G., Harrison S. C. (2018). Conserved
Epitope on Influenza-Virus Hemagglutinin Head Defined by a Vaccine-Induced
Antibody. Proc. Natl. Acad. Sci. U. S. A..

[57] Guthmiller J. J., Han J., Li L., Freyn A. W., Liu S. T. H., Stovicek O., Stamper C. T., Dugan H. L., Tepora M. E., Utset H. A. (2021). First Exposure to the Pandemic H1n1 Virus Induced Broadly Neutralizing
Antibodies Targeting Hemagglutinin Head Epitopes. Sci. Transl. Med..

[58] Zhou X., Wu Y., Zhu Z., Lu C., Zhang C., Zeng L., Xie F., Zhang L., Zhou F. (2025). Mucosal Immune Response in Biology,
Disease Prevention and Treatment. Signal Transduction
Targeted Ther..

[59] Okuya K., Yoshida R., Manzoor R., Saito S., Suzuki T., Sasaki M., Saito T., Kida Y., Mori-Kajihara A., Kondoh T. (2020). Potential
Role of Nonneutralizing Iga Antibodies in
Cross-Protective Immunity against Influenza a Viruses of Multiple
Hemagglutinin Subtypes. J. Virol..

[60] Sano K., Saito S., Suzuki T., Kotani O., Ainai A., van Riet E., Tabata K., Saito K., Takahashi Y., Yokoyama M. (2021). An Influenza
Ha Stalk Reactive Polymeric Iga
Antibody Exhibits Anti-Viral Function Regulated by Binary Interaction
between Ha and the Antibody. PLoS One.

[61] Lucas E. D., Huggins M. A., Peng C., O’Connor C., Gress A. R., Thefaine C. E., Dehm E. M., Kubota Y., Jameson S. C., Hamilton S. E. (2024). Circulating Klrg1­(+)
Long-Lived Effector
Memory T Cells Retain the Flexibility to Become Tissue Resident. Sci. Immunol..

[62] Monne I., Fusaro A., Nelson M. I., Bonfanti L., Mulatti P., Hughes J., Murcia P. R., Schivo A., Valastro V., Moreno A., Holmes E. C., Cattoli G. (2014). Emergence
of a Highly
Pathogenic Avian Influenza Virus from a Low-Pathogenic Progenitor. J. Virol..

[63] Beerens N., Heutink R., Harders F., Bossers A., Koch G., Peeters B. (2020). Emergence and Selection
of a Highly Pathogenic Avian
Influenza H7n3 Virus. J. Virol..

[64] Gerloff N. A., Khan S. U., Balish A., Shanta I. S., Simpson N., Berman L., Haider N., Poh M. K., Islam A., Gurley E. (2014). Multiple
Reassortment Events among Highly Pathogenic
Avian Influenza a­(H5n1) Viruses Detected in Bangladesh. Virology.

[65] Pu J., Yin Y., Liu J., Wang X., Zhou Y., Wang Z., Sun Y., Sun H., Li F., Song J. (2021). Reassortment
with Dominant Chicken H9n2 Influenza Virus Contributed to the Fifth
H7n9 Virus Human Epidemic. J. Virol..

[66] Song J. M., Van Rooijen N., Bozja J., Compans R. W., Kang S. M. (2011). Vaccination
Inducing Broad and Improved Cross Protection against Multiple Subtypes
of Influenza a Virus. Proc. Natl. Acad. Sci.
U. S. A..

[67] Wang Q., Yu J., Kadungure T., Beyene J., Zhang H., Lu Q. (2018). Armms as a
Versatile Platform for Intracellular Delivery of Macromolecules. Nat. Commun..

[68] Ma Y., Wang Y., Dong C., Gonzalez G. X., Song Y., Zhu W., Kim J., Wei L., Wang B. Z. (2022). Influenza Np Core
and Ha or M2e Shell Double-Layered Protein Nanoparticles Induce Broad
Protection against Divergent Influenza a Viruses. Nanomedicine.

[69] World Health Organization Serological Detection of Avian Influenza a(H7n9) Virus Infections by Modified Horse Red Blood Cells Haemagglutination-Inhibition Assay; https://www.who.int/publications/m/item/serological-detection-of-avian-influenza-a(h7n9)-virus-infections-by-modified-horse-red-blood-cells-haemagglutination-inhibition-assay. (Accessed 20 March 2026).

[70] Dong C., Wang Y., Zhu W., Ma Y., Kim J., Wei L., Gonzalez G. X., Wang B. Z. (2022). Polycationic Ha/Cpg Nanoparticles
Induce Cross-Protective Influenza Immunity in Mice. ACS Appl. Mater. Interfaces.

