# Supplementary material for: Mosaic Inverted Hemagglutinin Extracellular Vesicle Vaccines Elicit Protective Systemic and Mucosal Immunity against Heterosubtypic Influenza Infection
Source: ACS Nano. 2026 Apr 1;20(14):10858–71. doi: 10.1021/acsnano.5c13363 (PMC13085848; doi:10.1021/acsnano.5c13363)
Supplement: Supplementary file 1 [file nn5c13363_si_001.pdf]

Supporting information

Mosaic Inverted Hemagglutinin Extracellular Vesicle Vaccines Elicit Protective Systemic and Mucosal Immunity against Heterosubtypic Influenza Infection

Wandi Zhu[1], Lai Wei[1], Chunhong Dong[1], Joo Kyung Kim[1], Madeline Bruhn[1], Yao Ma[1], Alex Ferrante[1], Arini Arsana[1], Priscilla Omotara[1], Sang-Moo Kang[1], Bao-Zhong Wang[1*]

[1]Center for Inflammation, Immunity & Infection, Institute for Biomedical Sciences, Georgia State University, Atlanta, GA 30303, USA.

*Dr. Bao-Zhong Wang, E-mail: bwang23@gsu.edu

Figure S1

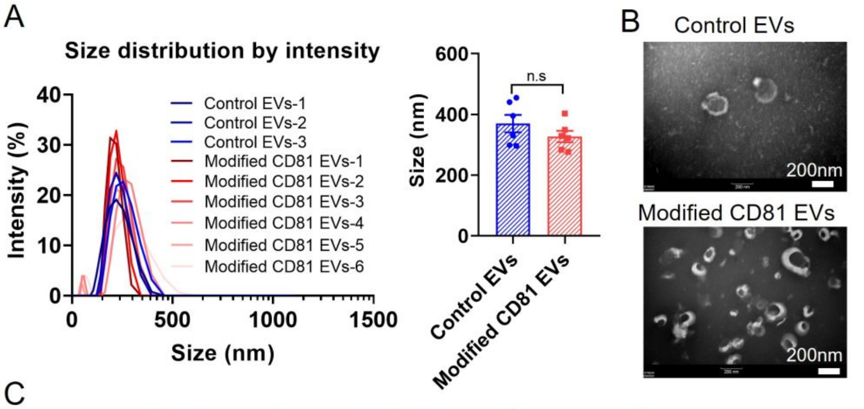

C

NTA	Test 1	Test 2	Test 3	Average	BCA (5E+07 cells supernatant)
Control EVs	1.43E+09	4.39E+08	1.06E+08	6.57E+08	155.2 μg
CD81 EVs	2.86E+09	5.55E+09	5.58E+09	4.66E+09	830 μg

Fig. S1. Overexpression of CD81 improved EV production. 293T cells were transfected with the CD81 plasmid, and EVs were harvested from the cultured supernatants three days post-transfection. The non-transfected cells were used as a control. (A) The sizes of EVs. (B) The observation of EVs by TEM. (C) Particle number and amount of purified EVs with and without CD81.

Figure S2

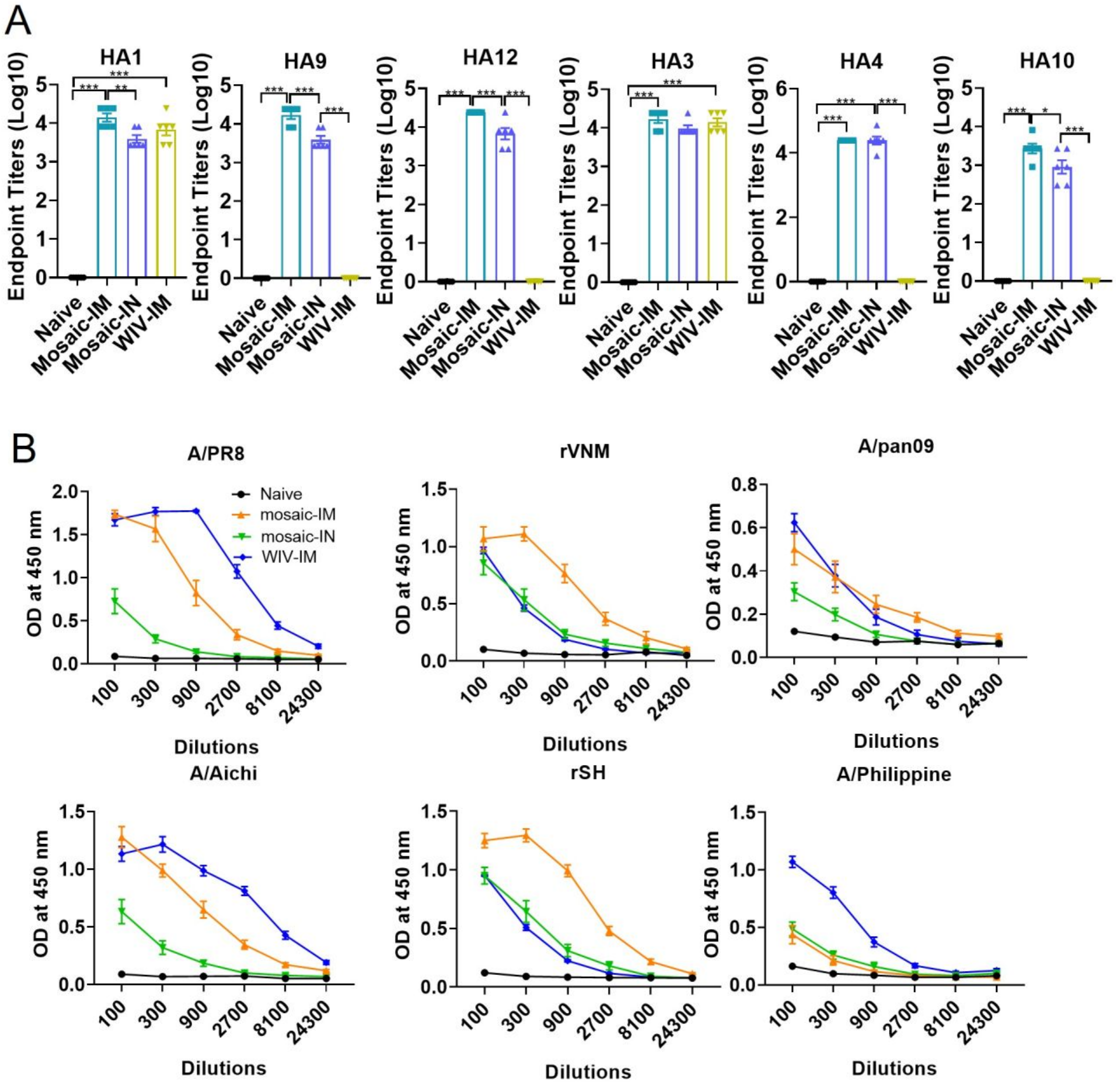

Figure S2. Different HA and virus-specific antibody responses after primary immunization. The recombinant proteins (HA10, HA4, HA3, HA9, HA12, and HA1) (A) and the influenza viruses (A/PR8, A/Aichi, A/pan09, A/Phi, rSH, and rVNM) (B) were used as coating antigens, respectively. The sera were serially diluted, and the antigen-specific IgG was analyzed by ELISA.

Figure S3

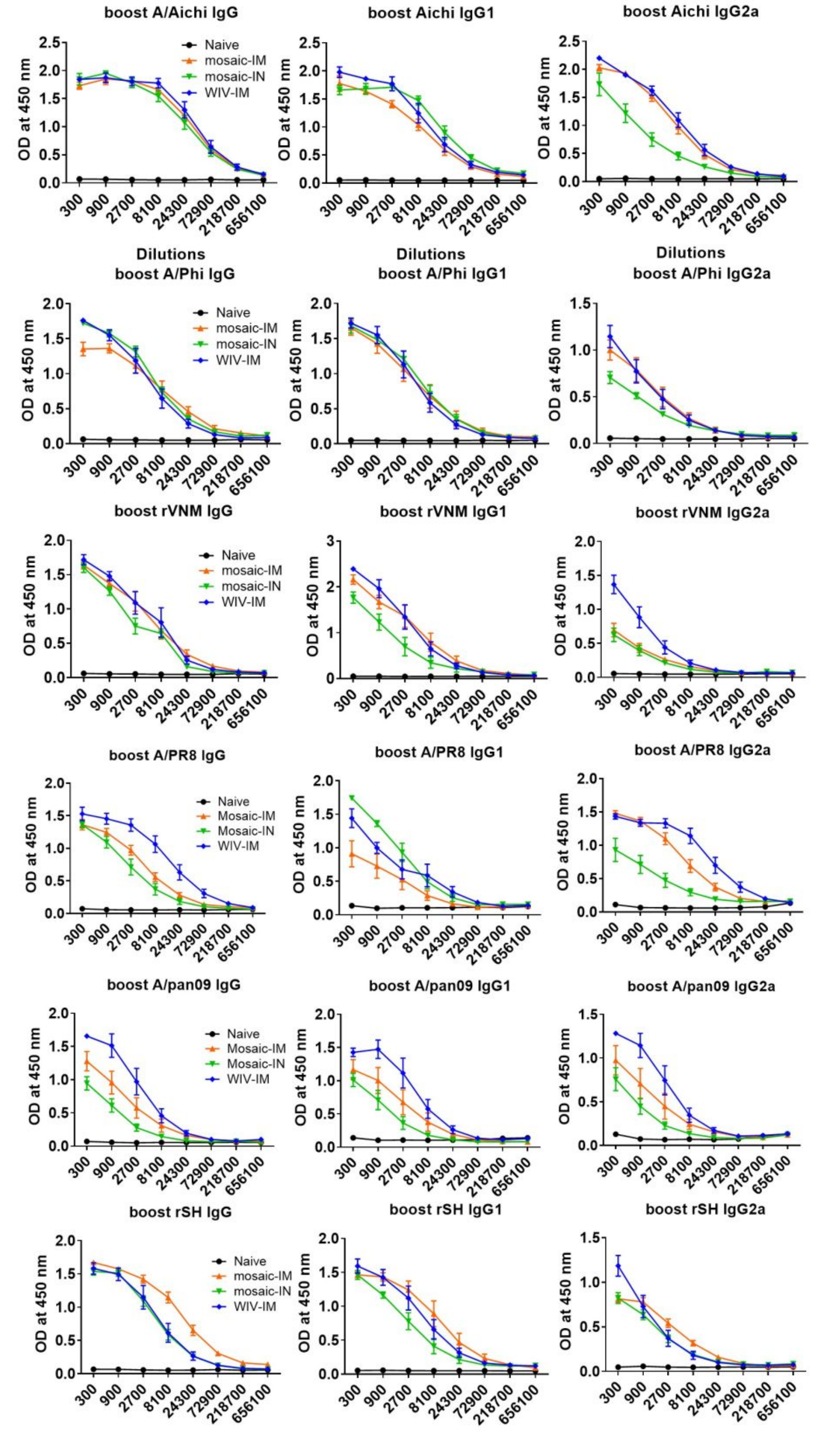

Figure S3. Virus-specific antibody responses after boosting immunization. Influenza viruses-A/PR8, A/Aichi, A/pan09, A/Phi, rSH, and rVNMs were used as coating antigens, respectively. The sera collected after boosting immunization were serially diluted to analyze antigen-specific IgG, IgG1, and IgG2a by ELISA.

Figure S4

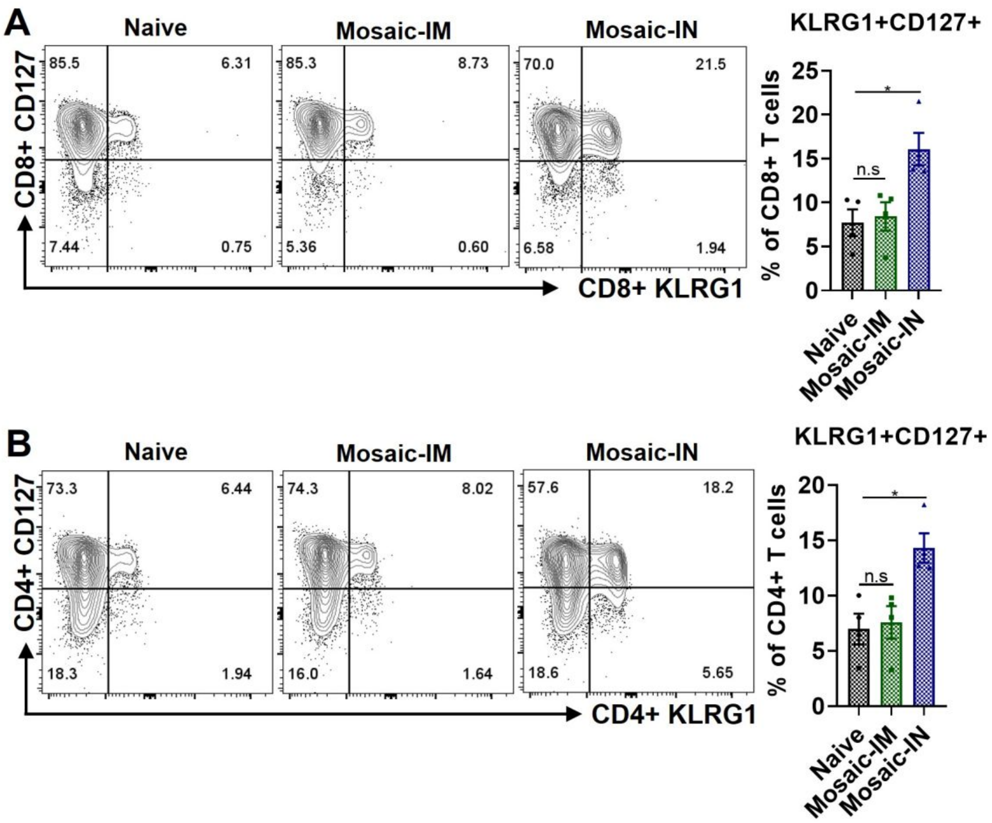

Figure S4. The expression of KLRG1 and CD127 on T cell populations. (A) and (B) KLRG1 and CD127 double positive CD8 and CD4 T cell population.

Figure S5

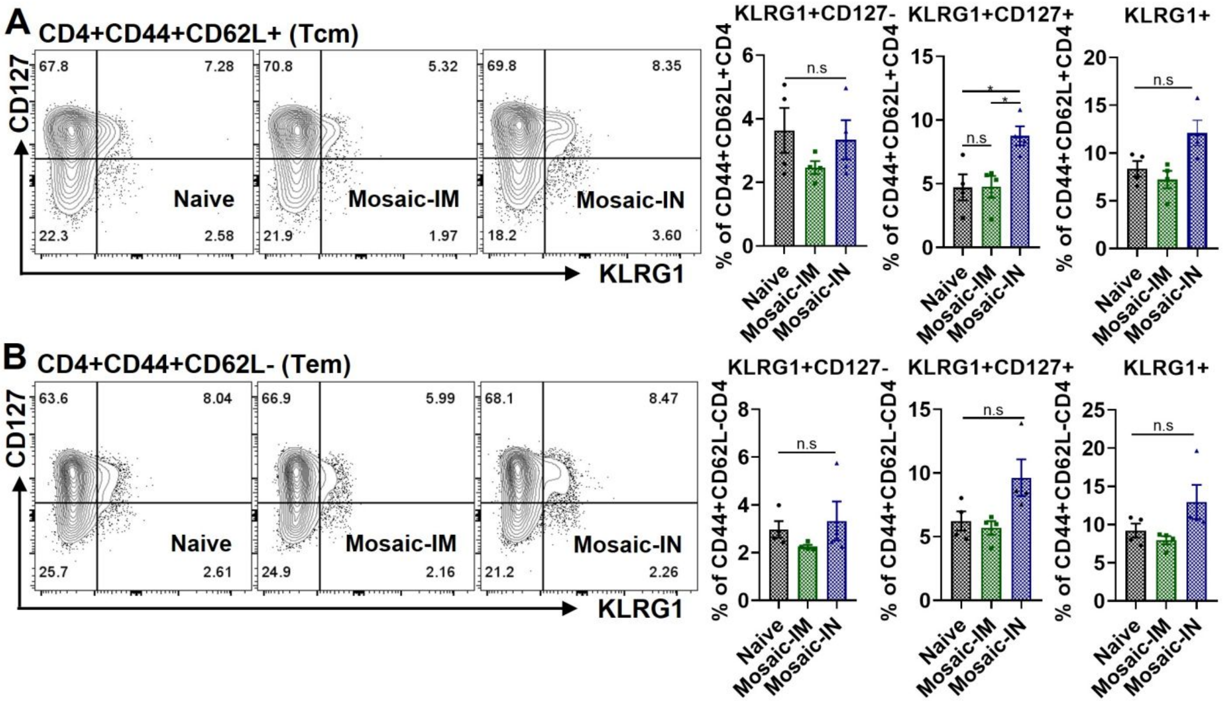

Figure S5. The expression of KLRG1 on CD4+ T cell populations. (A) and (B) The expression of KLRG1 and CD127 on CD4+ Tcm and CD4+ Tem populations.
